# ODANet: an occlusion and density aware network for small object detection of coffee cherry ripeness in complex field environments

**DOI:** 10.3389/fpls.2026.1745060

**Published:** 2026-03-30

**Authors:** Wenlong Yang, Jianan Song, Liya Li, Jianfeng Luo, Lihong Huang

**Affiliations:** Faculty of Intelligent Manufacturing Engineering, Guizhou Industry Polytechnic College, Guiyang, China

**Keywords:** attention mechanism, coffee cherry, deep learning, object detection, precision agriculture, ripeness assessment

## Abstract

**Introduction:**

Coffee cherry ripeness assessment is critical for harvesting efficiency and product quality, yet traditional manual inspection methods suffer from subjectivity and low efficiency.

**Methods:**

To address the challenges of detecting small, occluded, and densely distributed coffee cherries in complex field environments, this study proposes an Occlusion and Density Aware Network (ODANet). Built upon the YOLOv8 framework, ODANet integrates three innovative modules: (1) Condition-Guided Windowed Attention (CGWA), which incorporates occlusion and density maps as auxiliary guidance signals for efficient feature enhancement; (2) Attention-guided Space-Preserving Convolution (ASPC), which employs space-to-depth transformation with cascaded attention to preserve spatial information during downsampling; and (3) Dual-Adaptive Dynamic Upsampling (DADU), which achieves content-adaptive feature reconstruction through dual-branch offset prediction with learnable fusion weights.

**Results:**

Comprehensive evaluation on a publicly available dataset demonstrates that ODANet achieves state-of-the-art performance among 17 diverse detection architectures, attaining 76.7% mAP@0.5 with a 6.3 percentage point improvement over baseline YOLOv8, while maintaining computational efficiency (8.1 GFLOPs, 30.4M parameters) suitable for real-time deployment. Ablation studies validate the contributions of each module: ASPC improves performance by 2.2%, DADU by 0.6%, and CGWA by 3.5%.

**Discussion:**

The model demonstrates robust performance across varying lighting conditions, occlusion levels, and growth stages, making it particularly suitable for practical agricultural deployment. This research provides an efficient solution for small object detection in precision agriculture.

## 1 Introduction

Coffee represents one of the world’s most economically significant agricultural commodities, with global production fundamentally influencing rural livelihoods and international commerce (Godinho et al., 2022). Coffee cherry maturity assessment is critical for harvesting efficiency and product quality, directly impacting yield optimization and organoleptic characteristics (Andre et al., 2020; Bayman and Serrato-Diaz, 2025). Traditional methodologies rely on manual visual inspection—approaches that are labor-intensive, time-consuming, and susceptible to subjective interpretation, with assessment accuracy influenced by observer expertise, fatigue, and environmental conditions (Kazama et al., 2021). This subjective variability undermines consistency and introduces quality inconsistencies and economic inefficiencies in large-scale commercial production.

The advancement of computational methodologies has catalyzed a shift toward machine vision-based automated solutions for agricultural quality assessment, which have attracted increasing research attention as viable alternatives to manual evaluation (Xiao et al., 2023; Cano-Lara and Rostro‐González, 2024; Zhang, 2025). These computational frameworks leverage digital image processing, pattern recognition, and machine learning to enable rapid, objective, and efficient coffee cherry ripeness evaluation. By eliminating human subjectivity and enhancing processing throughput, these technologies offer innovative pathways for implementing scientific harvest management, improving product quality consistency, and minimizing economic losses from premature or delayed harvesting.

**Figure 1:**
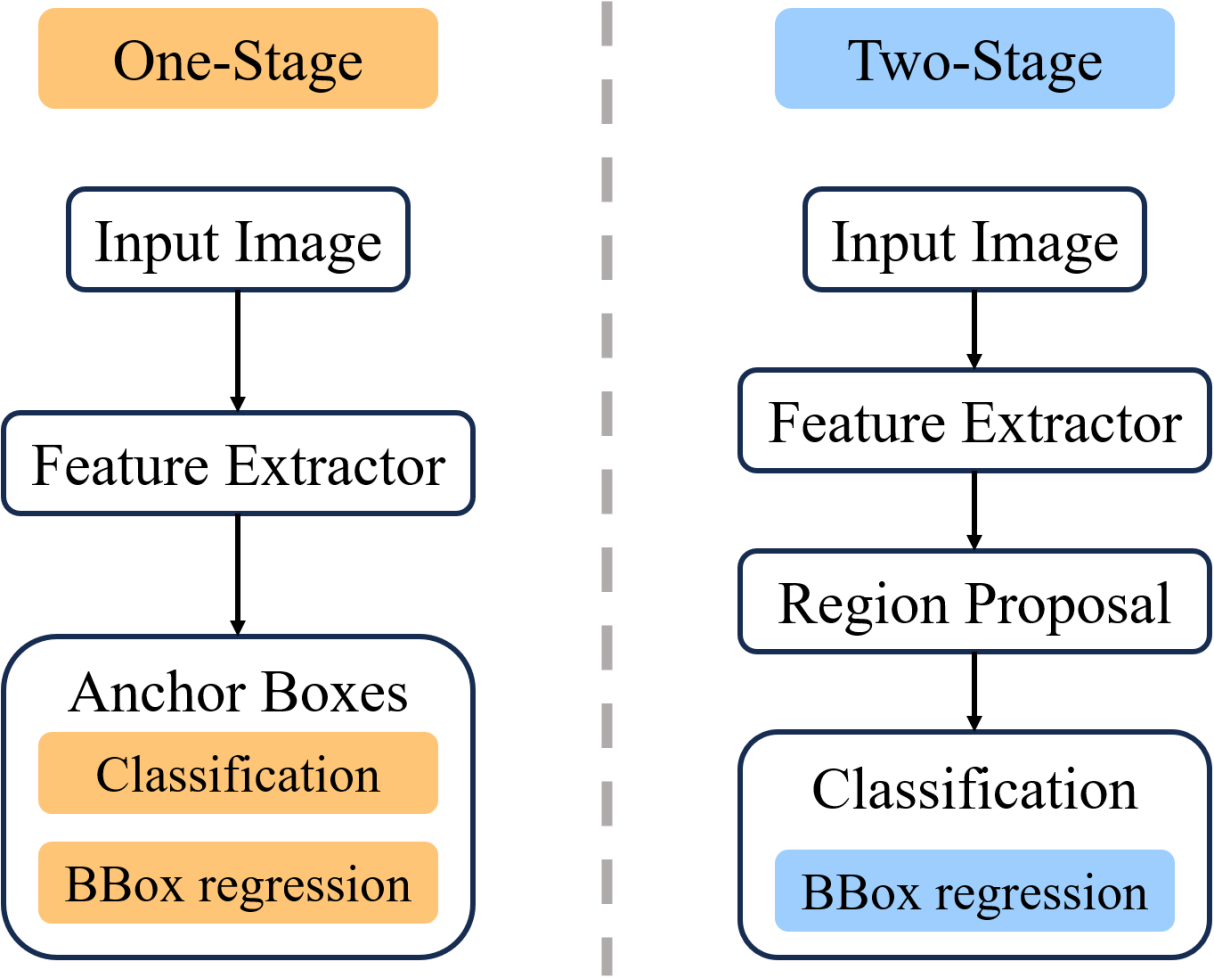
One -Stage vs Two-Stage detector workflow comparison.

Machine vision technology has evolved rapidly, transforming traditional manual inspection into mainstream automated quality control with widespread applicability across agricultural domains (Chen et al., 2002; Wang et al., 2022; Han et al., 2024). Within contemporary deep learning for computer vision, object detection has evolved along two principal paradigms: Two-Stage Detectors (Fast R-CNN, Faster R-CNN, Cascade R-CNN) employing hierarchical region proposal and refinement (Girshick, 2015; Ren et al., 2017; Cai and Vasconcelos, 2018), and One-Stage Detectors (YOLO (Redmon et al., 2016), RetinaNet (Lin et al., 2017), FCOS (Tian et al., 2019), and SSD (Liu et al., 2016)) directly predicting object locations prioritizing inference speed. Transformer-based architectures (DETR (Carion et al., 2020), Deformable DETR (Zhu et al., 2021)) introduced attention mechanisms modeling global relationships, while anchor-free approaches like CenterNet (Duan et al., 2024) represent objects through keypoint detection. Each architectural paradigm presents distinct trade-offs among detection accuracy, computational efficiency, and deployment complexity for agricultural applications as shown **Table 1** and **Figure 1**.

**Table 1:** Comparison of One-Stage and Two-Stage object detectors.

Taxonomy	Methods	Advantages	Disadvantages
Two-Stage	Fast R-CNN (Chen et al., 2002)	High accuracy	Slow detection speed
	Faster R-CNN (Ren et al., 2017)		
One-Stage	YOLO (Redmon et al., 2016)	Real-time	Moderate accuracy
	SSD (Liu et al., 2016)		

Given that coffee cherries are classified as small objects within detection taxonomies, they prove particularly susceptible to background complexity during recognition processes. Among contemporary detection frameworks, the YOLO architecture formulates detection as a unified regression task, enabling end-to-end training with exceptional speed supporting real-time requirements (Redmon et al., 2016). YOLO effectively leverages global image information to suppress background noise, demonstrates strong generalization capacity, and maintains lightweight architectural design suitable for embedded deployment (Tamayo-Monsalve et al., 2022). Based on these compelling advantages substantiated through extensive empirical validation, the present study adopts YOLOv8—representing the latest architectural iteration—as the foundational detection framework.

This research makes three primary contributions. First, we propose ODANet, integrating Condition-Guided Windowed Attention (CGWA), Attention-guided Space-Preserving Convolution (ASPC), and Dual-Adaptive Dynamic Upsampling (DADU) modules within YOLOv8 for enhanced small object detection in agricultural contexts. Second, we conduct comprehensive experiments against 17 detection architectures across multiple paradigms (two-stage, one-stage, transformer-based, anchor-free, and YOLO variants) under identical conditions. Third, we provide systematic ablation studies demonstrating each component’s contribution, offering valuable insights for lightweight deep learning architectures in precision agriculture.

## 2 Related work

### 2.1 Deep learning foundations in agricultural computer vision

Deep learning technology has established itself as the core methodological approach in agricultural computer vision research, primarily due to its capability to substantially accelerate inference processes while maintaining high detection accuracy standards (Liao and Tian, 2024). Compared with traditional image processing methodologies and conventional machine learning approaches, deep learning frameworks efficiently handle complex background environments, lighting variations, and morphological variability while achieving end-to-end automated feature extraction and decision-making capabilities (Liao and Tian, 2024). Recent years have witnessed extensive exploration of these technologies across various agricultural production stages, with particularly notable success in coffee cherry recognition and maturity assessment domains (Hu et al., 2018; Woo et al., 2018).

### 2.2 Object detection architectures in agricultural computer vision

Object detection has evolved through several distinct architectural paradigms. Two-stage detectors pioneered by Fast R-CNN (Girshick, 2015) and Faster R-CNN (Ren et al., 2017) introduced region proposal networks followed by classification refinement, achieving high accuracy at the cost of computational complexity. Subsequent improvements to Faster R-CNN include local keypoint-based enhancements (Ding et al., 2020) and Cascade R-CNN (Cai and Vasconcelos, 2018) which further improved localization through multi-stage refinement. One-stage detectors including YOLO (Redmon et al., 2016), RetinaNet (Lin et al., 2017) with focal loss addressing class imbalance, FCOS (Tian et al., 2019) eliminating anchor boxes, and SSD (Liu et al., 2016) demonstrated improved speed-accuracy balance. Recent transformer-based approaches like DETR (Carion et al., 2020) employ set prediction for end-to-end detection, while Deformable DETR (Zhu et al., 2021) improves computational efficiency through deformable attention. Anchor-free methods such as CenterNet (Duan et al., 2024) represent objects through center points and dimensions, simplifying detection pipelines. However, these diverse architectures exhibit varying performance characteristics for small object detection in complex agricultural environments.

### 2.3 Coffee cherry detection technologies

Within coffee cherry detection, Tamayo-Monsalve et al. developed a CNN-based classification model achieving accuracy exceeding 98% (Tamayo-Monsalve et al., 2022). Bazame et al. proposed a comprehensive maturity detection scheme based on YOLOv4, integrating high efficiency, low computational cost, and high precision (Bazame et al., 2022). Pawłowski et al. utilized YOLOv8 to develop a lightweight model for coffee seed detection (Pawłowski et al., 2024), while Kazama et al. demonstrated enhanced performance monitoring coffee fruit maturity using an improved CNN architecture (Kazama et al., 2024). Sha et al. developed an accurate quality inspection system for solder joints using fine-tuned YOLOv5 models (Sha et al., 2023). Beyond coffee, deep learning has been successfully applied to maturity assessment of other fruits including papaya (Behera et al., 2020) and citrus (Chen et al., 2022), demonstrating the generalizability of computer vision approaches across different agricultural commodities.

### 2.4 Attention mechanisms and small object detection

Attention mechanisms have emerged as pivotal technologies for enhancing deep learning performance in computer vision (Guo et al., 2022; Brauwers and Frasincar, 2023). However, some attention approaches incur prohibitively high computational costs (Vaswani et al., 2017). The non-local attention mechanism can capture long-range dependencies but with quadratic complexity (Wang et al., 2018). The Criss-Cross Attention Network employs decomposed attention achieving linear complexity (Huang et al., 2019) yet still generates considerable computational load. Small object detection represents a particularly challenging subdomain, as small objects occupy minimal pixel areas and are easily affected by background noise, occlusion, and limited feature representation (Jiang et al., 2020; Liao and Tian, 2024). Recent advances have explored feature pyramid networks, multi-scale fusion, and specialized attention mechanisms (Wang et al., 2022; Rasheed and Zarkoosh, 2025; Ye et al., 2025). This research addresses identified gaps by proposing ODANet, which synergistically integrates CGWA for efficient spatial reasoning, ASPC for information-preserving downsampling, and DADU for adaptive upsampling within the YOLOv8 framework.

## 3 Methods

### 3.1 Overall architecture

Traditional object detection architectures struggle to simultaneously handle occluded objects and densely distributed targets in complex agricultural environments, where overlapping leaves, branches, and clustered fruits significantly degrade detection performance.

**Figure 2:**
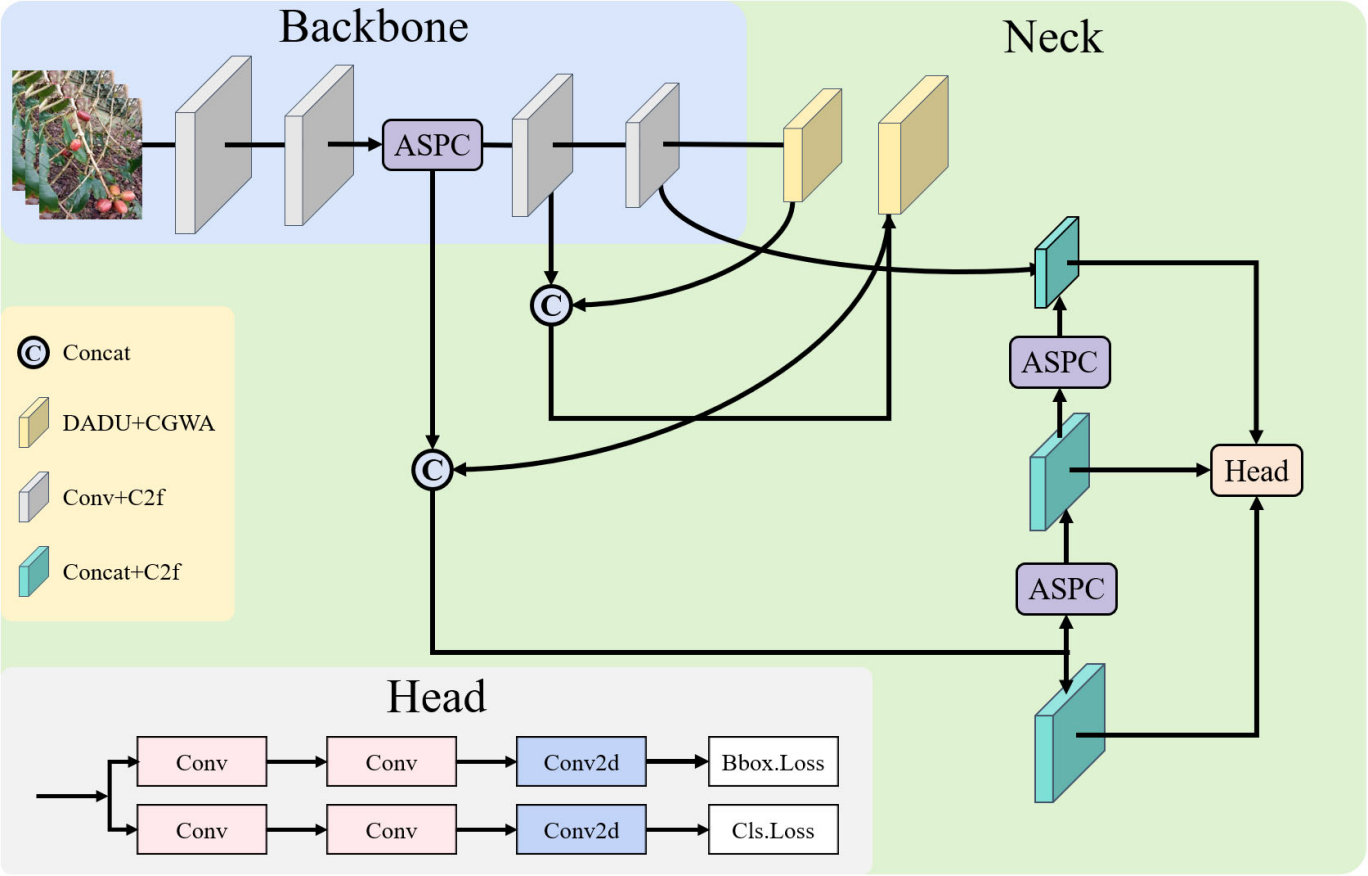
Overall architecture of ODANet.

**Figure 2** illustrates the overall architecture of ODANet, which builds upon the YOLOv8 framework with three specialized modules strategically integrated at critical stages of the detection pipeline. The backbone network extracts hierarchical features through progressive downsampling, with ASPC modules replacing conventional convolution layers to preserve spatial information during feature compression. The neck employs a Path Aggregation Network (PAN) structure enhanced with CGWA modules for condition-guided feature refinement, utilizing occlusion and density maps to provide auxiliary supervision signals. Finally, DADU modules replace traditional upsampling operations in the decoder to achieve content-adaptive feature reconstruction with improved spatial precision.

### 3.2 Condition-guided windowed attention

Field investigation in coffee orchards revealed that a substantial proportion of cherries are partially occluded by overlapping leaves and branches, particularly in lower canopy regions, while others appear in densely clustered formations along fruit-bearing nodes. Statistical sampling across 200 randomly selected images indicated that approximately 40% of cherries exhibit more than 30% visible-area occlusion, and nearly 35% are located within high-density regions where inter-fruit distances are below 15 pixels at 640×640 resolution. Under such conditions, feature responses are easily diffused into background foliage, leading to missed or incomplete detections.

**Figure 3:**
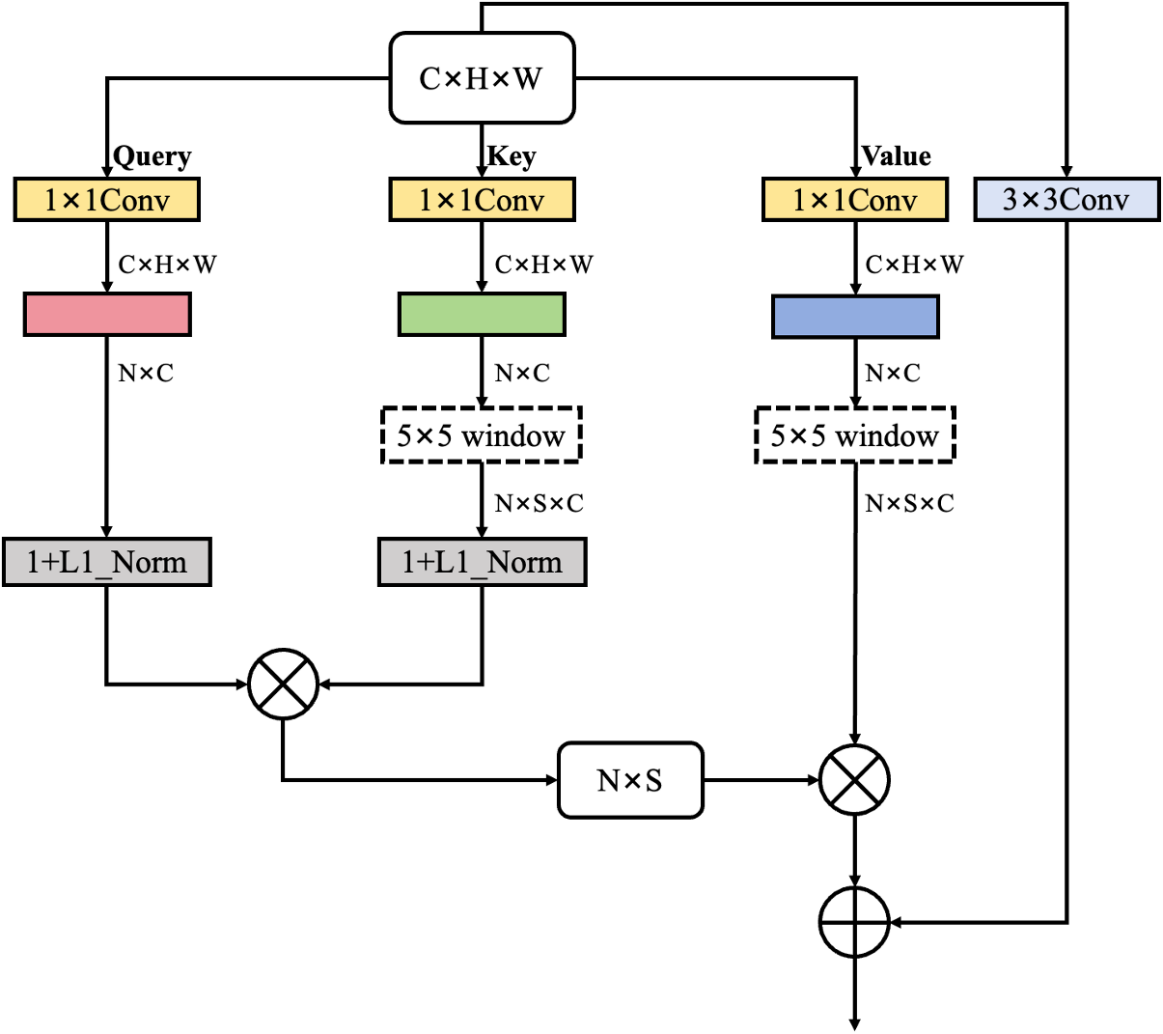
Architecture of the Condition-Guided Windowed Attention (CGWA) module.

Conventional attention mechanisms fail to effectively extract discriminative features from partially visible objects in scenarios with severe occlusion and dense spatial distributions, as they lack explicit guidance to focus on challenging regions where enhanced processing is most needed. In baseline YOLOv8, spatial attention operates uniformly across feature maps without awareness of canopy-induced visibility variation or fruit clustering intensity, resulting in diluted feature emphasis for partially visible cherries. To address this limitation, we propose Condition-Guided Windowed Attention (CGWA), which integrates multi-condition guidance with adaptive window-based self-attention to strengthen feature representations in regions affected by occlusion and density variation, as illustrated in **Figure 3**.

The module introduces dual condition encoding using occlusion and density maps as auxiliary guidance signals. The occlusion map is derived through local contrast attenuation and edge discontinuity analysis, which approximate visibility degradation caused by canopy overlap. The density map is generated via Gaussian kernel aggregation centered on annotated fruit locations during training, modeling spatial clustering intensity. The occlusion encoder employs a lightweight three-layer convolutional network to extract hierarchical occlusion features, capturing both local boundary transitions and broader occlusion patterns. In parallel, the density encoder processes density distributions to identify spatial regions requiring enhanced attention allocation. The density maps were generated using an adaptive Gaussian kernel method specifically designed for densely distributed, small-scale coffee cherry targets. The kernel parameters were dynamically adjusted based on the Euclidean center-to-center distances between adjacent cherry instances. The density maps were derived from manually annotated bounding-box coordinates in the coffee cherry dataset and generated via an offline preprocessing procedure. Each density map was maintained at a spatial resolution of 640×640 pixels, consistent with the input images, and all pixel values were normalized to the interval (0, 1) to reduce the effects of illumination variability.

The core attention computation operates within local windows of configurable size, typically configured as 7×7 spatial extents, which constrains the receptive field to capture fine-grained local dependencies while maintaining computational tractability. For input features of shape B×H×W×C, the module applies learned linear projections to generate query, key, and value representations following standard multi-head attention formulations. However, rather than computing attention globally across the entire spatial extent, the mechanism partitions the feature map into non-overlapping windows and performs self-attention independently within each partition. This windowed strategy reduces computational complexity from O((H×W)²) to O(Ws^4^×(H/Ws) ×(W/Ws)), where Ws denotes the window size, enabling efficient processing of high-resolution feature maps without prohibitive memory requirements.

To capture relative positional information within each window, the module employs learnable relative position bias tables that encode spatial relationships between all position pairs within a window. For a window size of Ws, the bias table contains (2Ws-1)² entries corresponding to all possible relative spatial displacements between positions. During attention computation, these bias terms are added to the attention logits prior to softmax normalization as shown in Equation 1:

(1) $$Attention\;\left(Q,\;K,\;V\right)\;=\;softmax\left(\left(QK^{T}/\sqrt{d_{k}}\right)\;+\;B\right)\;V$$

where B represents the relative position bias retrieved from the precomputed table using position-specific indices, and d_k_ denotes the dimensionality of key vectors. This relative encoding preserves local spatial structure while maintaining translation equivariance within window boundaries.

The adaptive modulation mechanism represents a critical innovation that dynamically adjusts attention weights based on window-level occlusion and density statistics derived from the condition maps. For each attention window, the module computes average occlusion and density scores through spatial pooling over the corresponding condition map regions. These aggregated scores are concatenated and processed through a compact feedforward network comprising two linear layers with ReLU activation and dropout regularization, which learns to predict head-specific modulation factors constrained to the range (0,1). The predicted modulation factors are applied multiplicatively to the attention logits, effectively amplifying attention weights in windows exhibiting high occlusion or density scores while maintaining standard processing in simpler regions. This adaptive behavior enables the model to automatically allocate enhanced representational capacity to challenging areas without requiring manual configuration or region-specific tuning.

Furthermore, the module incorporates a boundary enhancement network that identifies object boundaries and transition zones between occluded and visible regions. This auxiliary network processes concatenated occlusion and density maps through a two-layer convolutional architecture with sigmoid activation, generating a single-channel boundary weight map that highlights regions exhibiting sharp gradients in condition patterns. The boundary weights provide additional additive bias to attention logits in these critical edge regions, ensuring that the model maintains sensitivity to boundary information that is often degraded under occlusion. Following windowed attention computation across all spatial partitions, the module applies output projection and residual connection to produce the final enhanced feature representation.

The complete formulation of the conditioned attention mechanism can be expressed as shown in Equation 2:

(2) $$A_{enh}\;=\;softmax\left(\alpha \left(O,D\right)\cdot \left(QK^{T}/\sqrt{d_{k}}\right)\;+\;B\;+\;\beta \left(O,D\right)\right)\;V$$

where O and D represent occlusion and density conditions respectively, α (·) denotes the learned modulation function that scales attention based on condition severity, and β (·) captures boundary enhancement weights that emphasize edge information. This formulation explicitly conditions the attention mechanism on local spatial context, enabling adaptive feature enhancement that responds intelligently to scene-specific characteristics.

### 3.3 Attention-guided space-preserving convolution

Field observations indicate that immature and partially occluded coffee cherries frequently occupy fewer than 20×20 pixels in 640×640-resolution images, particularly under dense canopy regions. In such cases, early-stage feature compression significantly weakens small-object responses. Empirical feature visualization of baseline YOLOv8 revealed that several small green cherries lose distinguishable activation after the first two downsampling stages, leading to missed detections in cluttered foliage backgrounds.

Standard convolutional downsampling operations using strided convolutions or pooling layers introduce substantial information loss that disproportionately affects small and occluded objects, as approximately 75% of spatial positions are discarded during each downsampling stage, potentially causing complete feature loss for small targets.

In object detection networks, spatial resolution reduction through conventional downsampling approaches creates a fundamental information preservation challenge that becomes particularly severe for small and partially occluded objects common in agricultural scenarios. When reducing spatial resolution by a factor of two, traditional strided convolutions or pooling operations discard roughly three-quarters of the spatial positions, which can lead to complete loss of features for small objects whose spatial extent already approaches the receptive field of individual feature map locations. This information loss intensifies in agricultural detection tasks where target objects frequently exhibit small scales, irregular shapes, and partial occlusion patterns that make feature preservation critical for successful detection.

**Figure 4:**
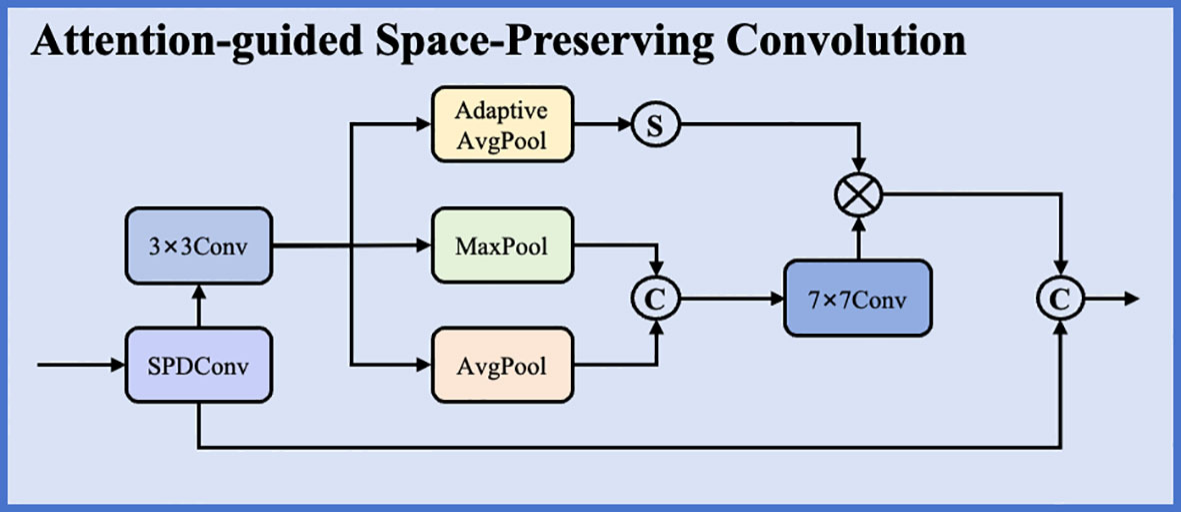
Architecture of the Attention-guided Space-Preserving Convolution (ASPC) module.

To address this fundamental limitation, we propose an Attention-guided Space-Preserving Convolution (ASPC) module that integrates space-to-depth transformation with cascaded attention mechanisms to maintain complete spatial information while performing feature refinement as shown in **Figure 4**. The module operates through three sequential stages: space-to-depth transformation for lossless spatial rearrangement, convolution-based feature processing, and dual-pathway attention for adaptive channel and spatial enhancement. The space-to-depth (SPD) transformation reorganizes spatial information into the channel dimension without discarding any data, converting input features of shape B×C×H×W into B×(4C) ×(H/2) ×(W/2) by rearranging non-overlapping 2×2 spatial blocks into four separate channel groups. This rearrangement preserves all spatial information while enabling subsequent convolutions to process a more compact feature representation.

Following the SPD transformation, the module applies a 3×3 convolution to extract spatial patterns across the rearranged features. This convolution operates on the expanded channel dimension, enabling the network to learn relationships between the reorganized spatial positions while maintaining complete information preservation. The convolution output then passes through two cascaded attention mechanisms that adaptively enhance feature representations based on learned importance weights.

The spatial attention pathway computes position-wise attention weights by processing the features through average pooling and max pooling along the channel dimension, followed by a 7×7 convolution and sigmoid activation. This pathway identifies spatially important regions by analyzing feature statistics across channels, generating attention weights that emphasize positions containing discriminative object information while suppressing background regions. The spatial attention mechanism can be formulated as shown in Equation 3:

(3) $$Msp\;=\;\sigma \left(Conv_{7\times 7}\left(\left[AvgPool\left(F\right);\;MaxPool\left(F\right)\right]\right)\right)$$

where F represents the input features, σ denotes the sigmoid activation, and []; indicates channel-wise concatenation. The resulting spatial attention map Msp modulates the features through element-wise multiplication, enhancing important spatial locations.

The channel attention pathway aggregates spatial information through both average pooling and max pooling, producing two channel-wise descriptors that capture complementary statistical properties. These descriptors are processed independently through a shared multi-layer perceptron with a reduction ratio that compresses the channel dimension before expansion, enabling efficient learning of channel dependencies. The outputs from both descriptors are summed and passed through sigmoid activation to generate channel attention weights. The channel attention mechanism follows the formulation as shown in Equation 4:

(4) Mch = σ(MLP(AvgPool(F)) + MLP(MaxPool(F)))

where MLP represents the shared multi-layer perceptron with bottleneck architecture. The channel attention weights Mch are applied multiplicatively to emphasize informative channels while suppressing less relevant ones.

The complete ASPC module combines space-preserving transformation, feature convolution, and cascaded attention through the following formulation as shown in Equation 5:

(5) $$F_{out}\;=\;Mch\;⊙\;\left(Msp\;⊙\;Conv_{3\times 3}\left(SPD\left(F_{in}\right)\right)\right)$$

where F_in_ and F_out_ denote input and output features respectively, and ⊙ represents element-wise multiplication. This formulation explicitly preserves complete spatial information through the SPD transformation while learning to emphasize relevant features through cascaded attention mechanisms, providing both information preservation guarantees and adaptive feature enhancement capabilities essential for detecting small and occluded objects.

### 3.4 Dual-adaptive dynamic upsampling

Field-based error analysis revealed that baseline YOLOv8 frequently produces blurred object boundaries or merged bounding boxes when cherries appear in dense clusters or under partial canopy shadow. In high-density fruiting nodes, adjacent cherries are occasionally reconstructed as a single elongated detection due to insufficient spatial refinement during feature upsampling. Moreover, illumination variation between sunlit and shaded regions alters local texture contrast, increasing reconstruction ambiguity in conventional upsampling stages.

Traditional upsampling methods such as bilinear interpolation and transposed convolution are limited by their content-agnostic or static nature, failing to adapt to varying object appearances caused by occlusion, lighting conditions, and growth stages in agricultural environments, which require flexible, content-dependent upsampling strategies.

Feature upsampling constitutes a critical operation in multi-scale object detection architectures, responsible for recovering spatial resolution lost during downsampling stages while propagating semantic information from coarse-level features to fine-level predictions. Traditional approaches exhibit fundamental limitations: bilinear interpolation applies fixed, content-agnostic interpolation kernels that ignore semantic structure and object boundaries, while transposed convolution learns static upsampling patterns during training but cannot adapt to varying input content at inference time. These limitations are particularly detrimental in orchard environments where fruit clustering and canopy-induced shadowing introduce region-specific structural complexity.

**Figure 5:**
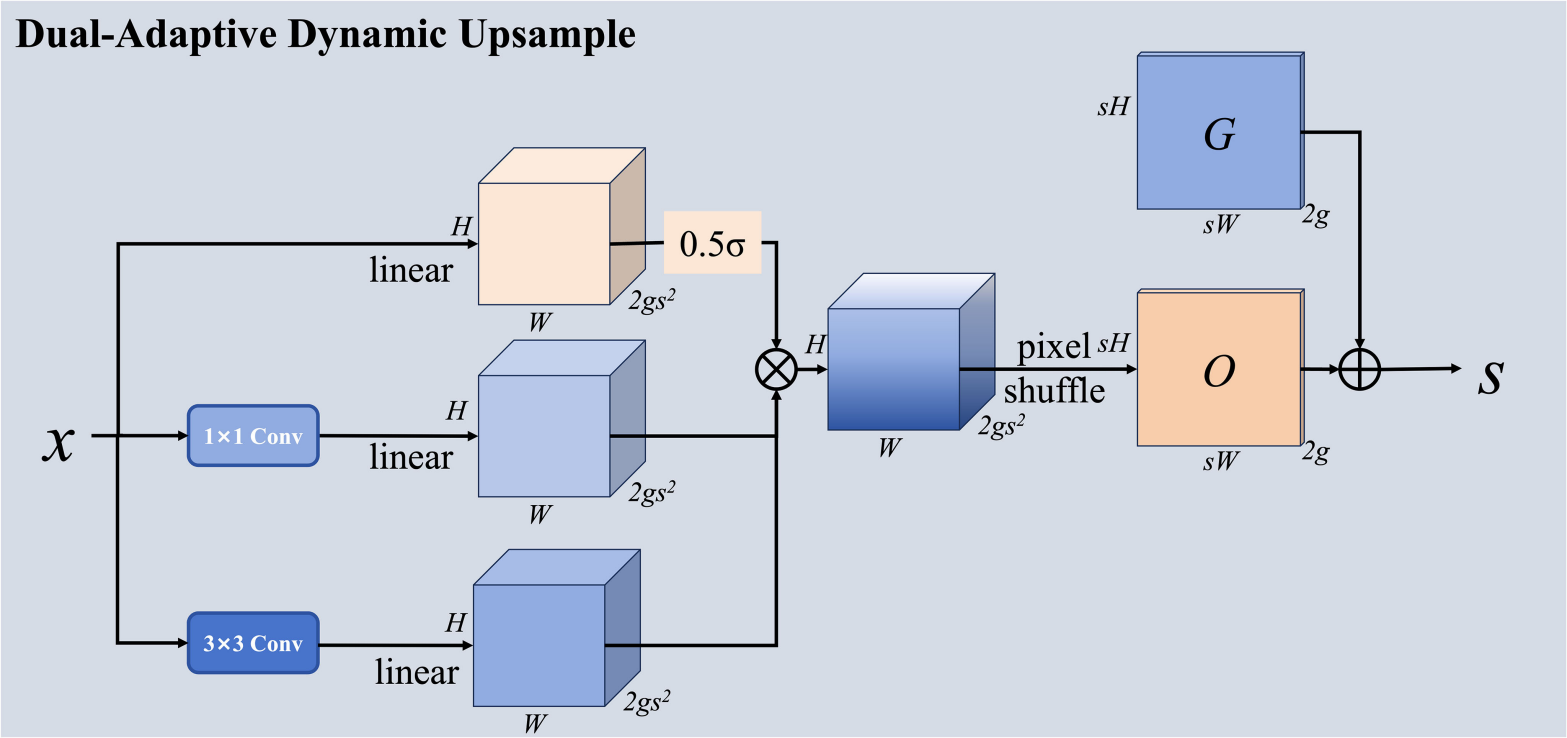
Architecture of the Dual-Adaptive Dynamic Upsampling (DADU) module showing du-al-branch offset prediction.

Recent dynamic approaches, such as DySample, introduce content-adaptive upsampling by predicting spatial offsets that modulate sampling positions instead of learning full convolution kernels, thereby improving flexibility with moderate computational cost. However, its single offset prediction pathway may struggle to simultaneously capture global structural consistency and fine-grained local boundary details. To address this limitation, we propose a Dual-Adaptive Dynamic Upsampling (DADU) module that generates offset predictions through two parallel branches with complementary receptive fields, as shown in **Figure 5**. The fused offsets are adaptively weighted through a learnable mechanism, enabling balanced modeling of global context and local structural variation.

The module processes input features X of shape B×C×H×W and produces upsampled outputs Y of shape B×C×(sH)×(sW), where s denotes the upsampling scale factor, typically set to 2 for incremental resolution recovery in standard detection architectures. The upsampling process consists of three sequential stages: optional pre-processing via pixel shuffle for initial resolution expansion, dual-branch offset prediction with learned fusion for content-adaptive sampling position generation, and dynamic sampling using the predicted offsets for final output generation.

When the configuration specifies the pixel-shuffle-then-linear (PL) mode, the module first applies a pixel shuffle operation to rearrange channel-to-space information, providing a coarse initial upsampling before refinement. For a scale factor s, pixel shuffle reorganizes input of shape B×(s²C) ×H×W into B×C×(sH)×(sW) by interpreting s² channel groups as spatial positions in the output grid. This pre-upsampling reduces the complexity of subsequent offset prediction by operating on already-expanded spatial dimensions, enabling the module to refine existing upsampled features rather than performing upsampling entirely through dynamic sampling.

The dual branch offset prediction architecture constitutes the core innovation of the proposed module, designed to capture complementary aspects of the upsampling transformation through parallel pathways with different receptive fields. The first branch employs a 1×1 convolution that operates independently at each spatial location without considering neighborhood context, processing each position based solely on its channel values. This pointwise convolution captures global channel dependencies and enables rapid offset computation with minimal computational cost, making it suitable for modeling consistent upsampling patterns that apply uniformly across the feature map regardless of local content variations. The second branch utilizes a 3×3 convolution with standard padding that aggregates information from 3×3 local neighborhoods, incorporating spatial context into the offset prediction process. This spatially aware pathway captures local texture details, structural patterns, and boundary information that require contextual reasoning, enabling content-adaptive offset generation that responds to local variations in object appearance, occlusion patterns, and semantic transitions.

Both branches independently predict offsets of shape B×(2Gs²) ×H×W, where G denotes the number of sampling groups used to partition channels for efficient processing, and the factor 2 accounts for horizontal and vertical offset components required to specify 2D sampling positions. The offset dimensionality s² stems from the requirement to predict offsets for each of the s² sub-pixel positions in the upsampled output grid. By partitioning the C channels into G groups, the module performs group-wise sampling that reduces computational complexity from O(C²) to O(C²/G) while maintaining sufficient modeling flexibility to capture diverse upsampling patterns across channel subsets. The two predicted offset fields, denoted as Δ_1_ and Δ_2_, capture complementary aspects of the upsampling transformation: Δ_1_ emphasizes global consistency and uniform patterns, while Δ_2_ captures local adaptivity and content-dependent variations.

To optimally combine these complementary predictions, the module employs a learnable scalar weight α initialized to 0.5, which controls the relative contribution of each branch through automatic adaptation during training. The sigmoid activation constrains α to the range (0,1), ensuring stable fusion dynamics and interpretable weight magnitudes that reflect the learned importance of each pathway. During training, this weight is updated through gradient descent to discover the optimal balance between global and local offset patterns for the specific detection task and dataset characteristics. The final fused offset field is computed as shown in Equation 6:

(6) $$\Delta fused\;=\;\sigma \left(\alpha \right)\;\cdot \;\Delta_{1}\;+\;\left(1\;-\;\sigma \left(\alpha \right)\right)\;\cdot \;\Delta_{2}$$

where σ denotes the sigmoid function applied to the learnable weight parameter. This weighted fusion enables automatic adaptation to different feature characteristics across spatial locations and semantic contexts, with the learned weight providing interpretable insight into the relative importance of global versus local information for effective upsampling.

The dynamic sampling operation uses the predicted offset field to generate the upsampled output through grid sampling with bilinear interpolation, which provides differentiable sampling required for end-to-end training. For each position (i,j) in the output feature map, the module computes the corresponding sampling position in the input by adding the base grid position to the predicted offset, then normalizes these coordinates to the range (–Godinho et al., 2022; Godinho et al., 2022) required by the grid sampling function. These normalized sampling coordinates are used to interpolate values from the input features using bilinear weights based on the four nearest neighbors. When dynamic scope is enabled, the module further modulates offset magnitudes through a learned scope factor predicted by an auxiliary 1×1 convolution, enabling the model to control the effective receptive field of the sampling operation and prevent excessive spatial deformation. The complete sampling operation can be expressed as shown in Equation 7:

(7) Y[b,:,i,j] = Σg Bilinear(X[b,g,:,:],Pbase[i,j] + Δfused[b,g,i,j])

where Pbase represents the regular grid positions defining the standard upsampling layout, g indexes the sampling groups that partition channels for efficient processing, and Bilinear (·) denotes bilinear interpolation with border padding mode to handle out-of-bounds coordinates that may result from large, predicted offsets. The group-wise sampling and averaging enables the module to capture diverse upsampling patterns across different channel subsets while maintaining computational efficiency through parallel processing.

The dual-branch architecture with learned fusion provides several key advantages over single-pathway alternatives that justify its increased complexity. First, the complementary receptive fields of 1×1 and 3×3 convolutions enable the module to simultaneously model both global upsampling trends that maintain structural coherence across the feature map and local content-dependent variations that adapt to object boundaries and semantic transitions. Second, the learnable fusion weight allows the model to automatically discover the optimal balance between these two aspects during training based on the specific characteristics of the dataset and task, eliminating the need for manual tuning or heuristic fusion rules that may not generalize across different scenarios. Third, the lightweight branch designs ensure that the additional modeling capacity comes with minimal computational overhead, making the module suitable for real-time detection applications where inference efficiency is critical. The complete formulation encapsulates content-adaptive upsampling with multi-scale offset prediction shown in Equation 8:

(8) $$DADUDual\left(X\right)\;=\;GridSample\left(X,\alpha \cdot Branch_{1}\left(X\right)\;+\;\left(1-\alpha \right)\cdot Branch_{2}\left(X\right)\right)$$

This formulation explicitly couples content-dependent offset prediction with dual-scale reasoning, enabling effective feature upsampling that adapts to local scene characteristics such as object density and occlusion patterns while maintaining global structural coherence necessary for accurate spatial localization of detected objects.

## 4 Experimental results and analysis

### 4.1 The experiments were conducted on a workstation equipped with an RTX4090 GPU and an Intel Core i7-14700KF CPU. The model was implemented under CUDA 12.1, trained using the SGD optimizer, and initialized with a learning rate of 0.01

In this study, coffee cherry maturity was quantitatively determined based on exocarp color, a physiologically reliable indicator of ripening progression in coffee cultivation. To ensure annotation reproducibility and eliminate subjective bias from manual grading, statistically derived thresholds in HSV and RGB color spaces were established using representative annotated samples. Unripe cherries were defined by dominant green coloration with HSV hue values of 60°–120°, saturation of 0.35–0.85, and RGB characteristics satisfying G > R and G > B. Semi-ripe cherries exhibited transitional yellow–orange tones with HSV hue values of 20°–60° and RGB features characterized by R ≈ G or R slightly greater than G, accompanied by increased brightness variance due to mixed pigment composition. Fully ripe cherries were identified by dominant deep red coloration with HSV hue values of 0°–20° or 340°–360°, saturation > 0.6, and empirically determined RGB thresholds of R > G + 15 and R > B + 15, corresponding to the 95% confidence interval of annotated ripe samples. This quantitative colorimetric framework enables objective, reproducible maturity classification for dataset construction and model training (Sudana et al., 2020).

The experimental validation used a publicly available coffee cherry ripeness dataset from Kaggle, comprising 4,320 high-resolution images captured under diverse orchard conditions. Each image contains manually annotated bounding boxes with categorical ripeness labels spanning three maturity stages: unripe (Tidak matang), semi-ripe (Setengah matang), and fully ripe (Matang). The dataset underwent preprocessing including normalization, resolution adjustment to 640×640 pixels, and quality filtering. Data augmentation incorporated geometric transformations (horizontal/vertical flipping, multi-scale resizing, rotation ±15°, random cropping) and photometric augmentations (brightness ±30%, contrast ±25%, hue ±10°, saturation ±30%).

**Figure 6:**
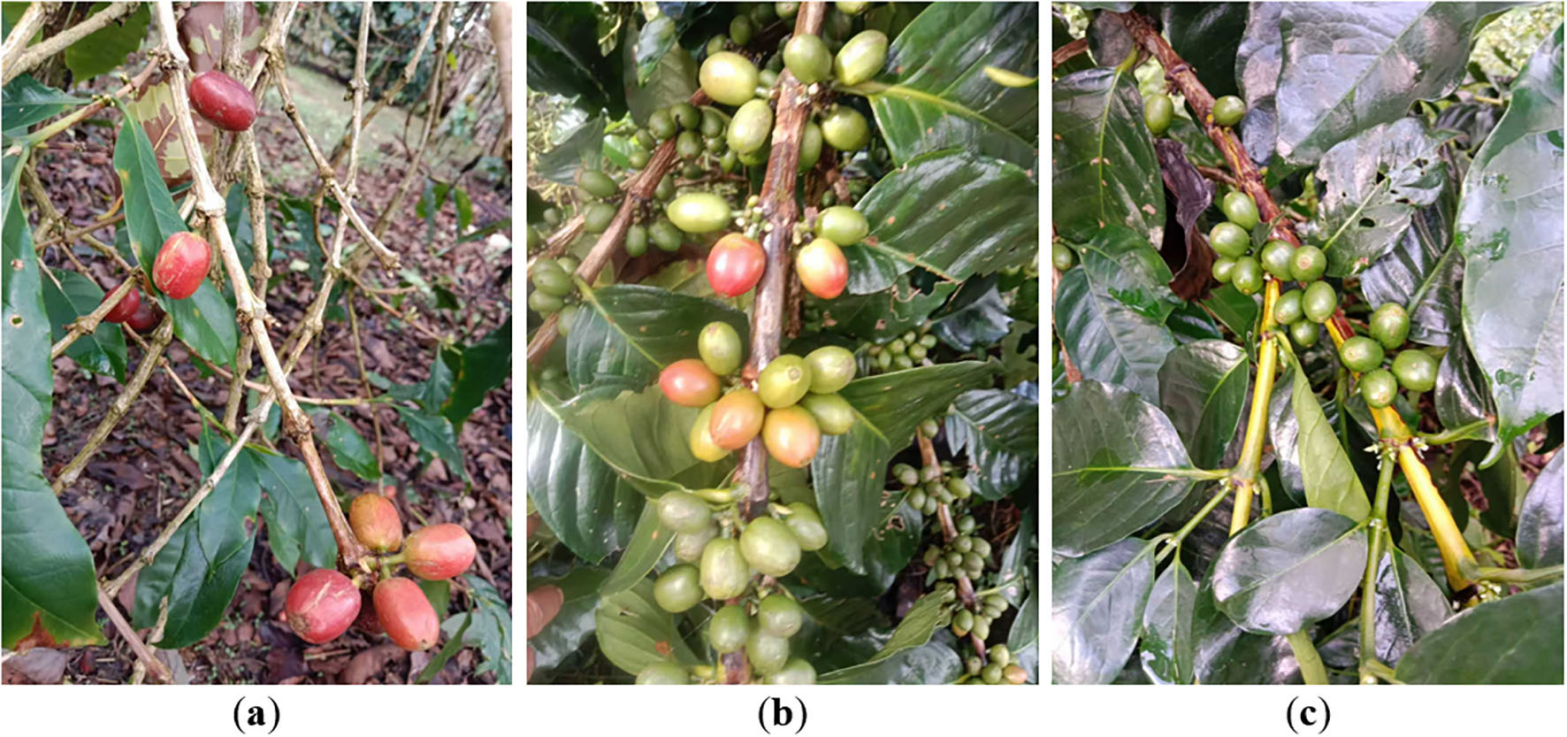
Image of coffee cherry. **(a)** Ripe; **(b)** Semi-ripe; **(c)** Unripe.

**Figure 7:**
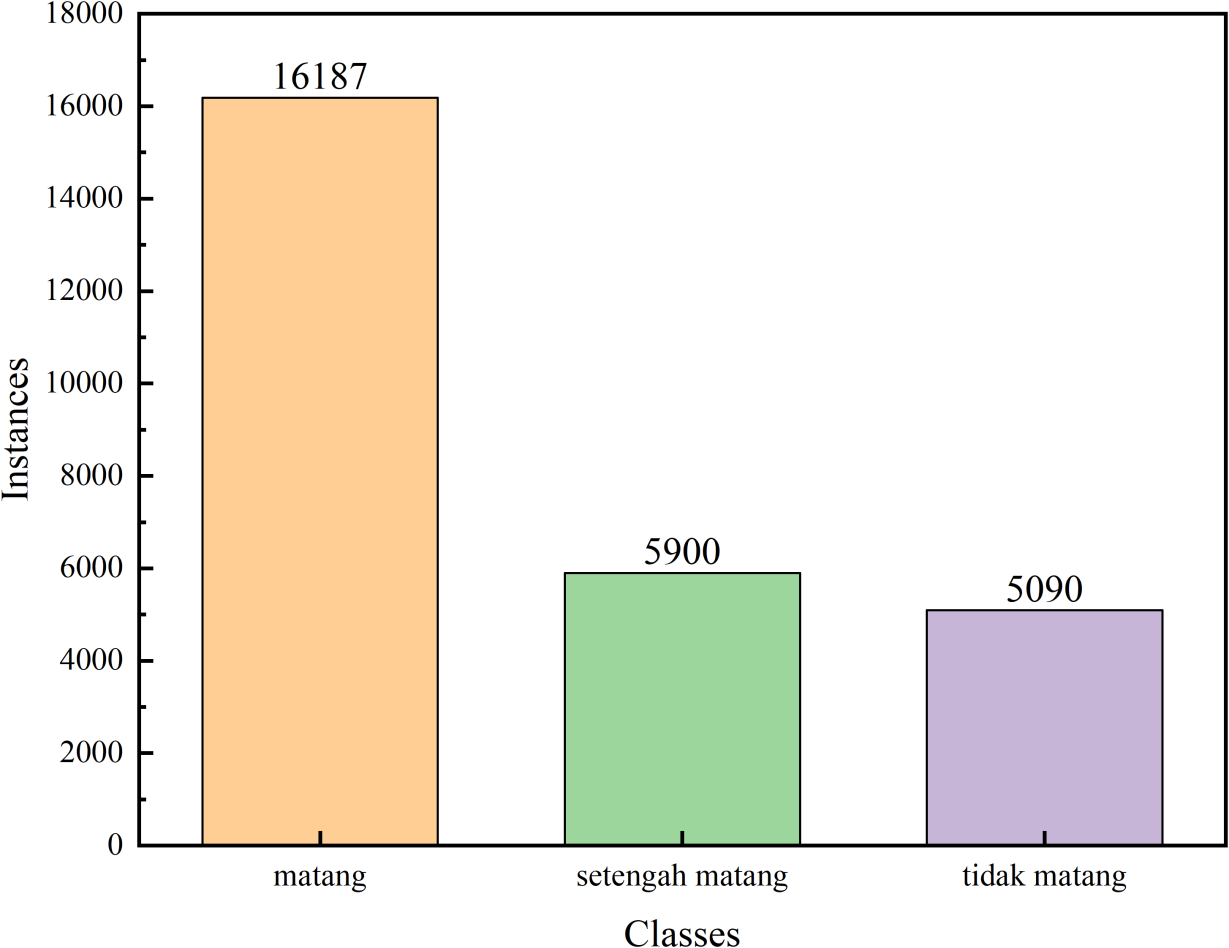
Statistical information on different types of bounding boxes.

**Figures 6**, **Figure 7** provide statistical analysis of the dataset’s bounding box distribution across three ripe categories. The histogram reveals class imbalance, with ripe cherries comprising the largest proportion, followed by semi-ripe and unripe categories. The size distribution shows coffee cherries predominantly fall into the small object category, with most bounding boxes occupying less than 32×32 pixels in the 640×640 resolution. This poses significant challenges as limited pixel coverage provides minimal visual information. The analysis also reveals substantial variation in object scales and aspect ratios.

The dataset was partitioned into training (70%), validation (15%), and testing (15%) subsets. Experiments were conducted on an NVIDIA RTX 3090 GPU with Intel Core i9-12900K processor and 64GB DDR5 memory, utilizing PyTorch 1.13.0 with CUDA 11.7, as shown in **Table 2**. Training employed AdamW optimizer with initial learning rate 0.001, cosine annealing scheduling, batch size 16, and 300 epochs with early stopping. Performance was evaluated using mAP@0.5, precision, recall, F1-score, inference latency, GFLOPs, and parameter count. All compared models were trained and evaluated under identical conditions to ensure fair comparison.

**Table 2:** Specific experimental setup.

Experimental configuration	Parameter	Experimental configuration	Parameter
GPU	RTX4090	CPU	14700KF
Batch size	16	Learning rate	0.01
CUDA	12.1	Optimizer	SGD
Epochs	300	Early Stopping	10

The evaluation of the model centers on two key metrics: detection speed and detection accuracy. Detection speed reflects the model’s inference efficiency measured in frames per second (FPS) and computational complexity quantified through GFLOPs, determining real-time deployment feasibility for field applications. Detection accuracy encompasses precision (proportion of correct positive predictions), recall (proportion of actual positives correctly identified), F1-score (harmonic mean balancing precision and recall), and mean average precision at IoU threshold 0.5 (mAP@0.5), which serves as the primary performance indicator for multi-class object detection by averaging precision values across all categories weighted by their prevalence.

### 4.2 Ablation study

To systematically elucidate individual contributions of each proposed module, we conducted comprehensive ablation experiments following a sequential additive protocol. Beginning with baseline YOLOv8, we incrementally integrated each enhancement module while maintaining all other factors constant.

The study of ablation demonstrates that each component makes essential contributions. The baseline YOLOv8 achieved 70.4% mAP@0.5. ASPC integration improved performance to 72.6%, adding 2.2%, validating effective spatial information preservation. Subsequently incorporating DADU enhanced performance to 73.2%, adding 0.6%. The complete ODANet architecture with all three modules achieved optimal performance at 76.7% mAP@0.5, achieving 6.3% cumulative improvement while maintaining computational efficiency at 8.1 GFLOPs and 30.4M parameters, as shown in **Table 3**.

**Table 3:** Ablation study of ODANet architecture.

ASPC	DADU	CGWA	mAP@0.5/%	Precision/%	Recall/%	FLOPS/G	Parameters/M
			70.4	76.4	65.9	8.2	30.1
✓			72.6	76.6	67.3	8.7	32.2
✓	✓		73.2	77.2	70.6	8.5	34.3
✓	✓	✓	76.7	77.5	75.3	8.1	30.4

**Figure 8:**
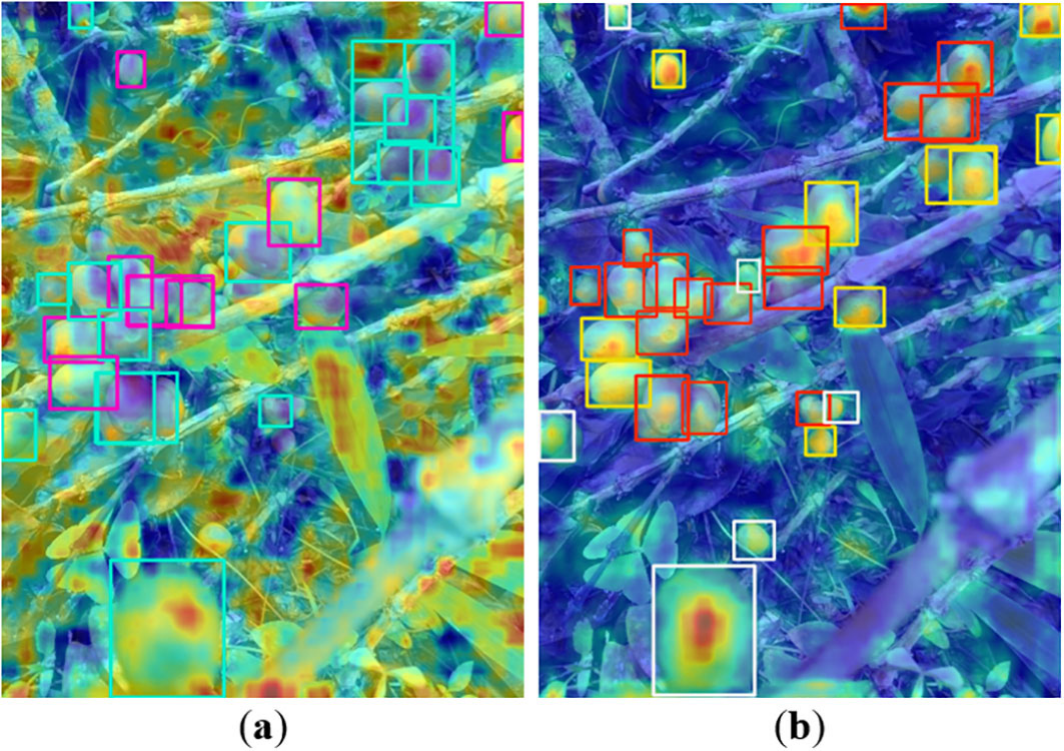
CAM visualization of the model. **(a)** Before improvement; **(b)** After improvement.

**Figure 8** presents Class Activation Mapping visualizations providing interpretable insights into the model’s attention mechanisms before and after architectural improvements. Image shows the baseline YOLOv8 model’s activation patterns, where attention is diffusely distributed across the image with limited discrimination between foreground coffee cherries and background foliage. The heatmap reveals that the baseline model struggles to focus on small target objects, with substantial activation intensity appearing in irrelevant background regions containing leaves and branches. In contrast, image b demonstrates the improved ODANet model’s activation patterns after integrating CGWA, ASPC, and DADU modules. The enhanced model exhibits significantly more concentrated attention on actual coffee cherry locations, with high-intensity activations precisely aligned with ground truth object positions. Background regions show substantially reduced activation, indicating improved discrimination capability. The CAM visualization validates that the proposed modules, particularly CGWA’s condition-guided attention, effectively guide the network to focus computational resources on relevant regions containing target objects while suppressing distracting background features. This improved feature localization directly translates to the observed quantitative performance gains in detection accuracy.

### 4.3 Component-wise ablation analysis

ASPC achieved substantially superior performance at 72.6% mAP@0.5, representing a 2.2 percentage point improvement over baseline and outperforming all alternatives. This validates ASPC’s space-to-depth transformation with cascaded attention for preventing information loss during downsampling, as shown in **Table 4**.

**Table 4:** Comparison of different downsampling methods.

Downsampling method	mAP@0.5/%	Parameters/M
Standard Convolution	70.4	30.1
Depthwise Separable	71.3	28.9
Max-Pooling	70.8	30.2
Average-Pooling	70.7	30.1
ASPC (Proposed)	72.6	32.2

**Table 5** presents a comprehensive comparison of different upsampling methods integrated into the YOLOv8 framework. The evaluation encompasses traditional approaches including nearest neighbor interpolation (70.3% mAP@0.5), bilinear interpolation (70.4% mAP@0.5), and transposed convolution (70.8% mAP@0.5), alongside advanced content-adaptive methods including CARAFE (71.2% mAP@0.5), DySample (71.5% mAP@0.5), and the proposed DADU module. Results demonstrate that traditional upsampling methods achieve similar baseline performance but lack content-adaptivity to handle diverse object appearances. CARAFE improves upon traditional methods through content-aware reassembly of features but incurs increased computational cost (9.2 GFLOPs). DySample achieves competitive performance with moderate computational requirements through dynamic offset prediction. The proposed DADU module attains optimal performance (71.9% mAP@0.5) while reducing computational cost to 8.1 GFLOPs, representing a 0.4 percentage point improvement over DySample with enhanced efficiency. This superior performance-efficiency trade-off stems from DADU’s dual-branch architecture with learned fusion, which enables the module to simultaneously capture global consistency through pointwise convolution and local content-dependent variations through spatial convolution, then optimally combine these complementary predictions through learned weights. The results validate that explicit modeling of multi-scale upsampling patterns through dual-branch offset prediction provides superior content-adaptive reconstruction compared to single-pathway alternatives.

**Table 5:** Comparison of different upsampling methods.

Upsampling method	mAP@0.5/%	FLOPS/G
Nearest Neighbor	70.4	8.2
CARAFE (Wang et al., 2022)	71.7	8.4
DADU (Proposed)	71.9	8.1

**Table 6** provides extensive comparison of attention mechanisms integrated into the YOLOv8 architecture. The evaluation includes coordinate attention (CA, 71.8% mAP@0.5, 29.7M parameters), squeeze-and-excitation attention (SE, 72.1% mAP@0.5, 29.8M parameters), convolutional block attention module (CBAM, 72.3% mAP@0.5, 29.9M parameters), efficient channel attention (ECA, 71.9% mAP@0.5, 29.6M parameters), and the proposed condition-guided windowed attention (CGWA, 73.4% mAP@0.5, 29.5M parameters). Traditional attention mechanisms achieve moderate performance improvements over baseline but lack explicit guidance to focus on challenging regions characterized by occlusion and high object density. CA effectively encodes position information but increases parameter count. SE provides channel-wise feature recalibration but may not sufficiently address spatial reasoning for small object detection. CBAM sequentially applies channel and spatial attention, achieving better performance than standalone mechanisms but still lacking scene-specific adaptivity. ECA simplifies SE through 1D convolution for channel attention while maintaining competitive performance with reduced parameters. The proposed CGWA module achieves optimal performance (73.4% mAP@0.5) with the smallest parameter count (29.5M parameters), representing improvements of 1.6, 1.3, 1.1, and 1.5% over CA, SE, CBAM, and ECA respectively. This superior performance stems from CGWA’s explicit incorporation of occlusion maps and density maps as auxiliary guidance signals that direct attention to challenging regions requiring enhanced representational capacity. The windowed attention mechanism with linear complexity enables efficient processing of high-resolution features without prohibitive computational cost, while adaptive modulation dynamically adjusts attention weights based on window-level occlusion and density statistics. These results conclusively demonstrate that condition-guided attention with explicit spatial reasoning outperforms generic attention mechanisms for agricultural small object detection in complex environments.

**Table 6:** Comparison of different attention mechanisms.

Attention	mAP@0.5/%	Parameters/M	FLOPS/G
YOLOv8-Base	70.4	30.1	8.2
SE (Hu et al., 2018)	71.4	30.4	8.2
CBAM (Woo et al., 2018)	71.5	30.3	7.9
ECA (Wang et al., 2020)	72.6	29.7	8.2
CA (Hou et al., 2021)	71.6	30.4	8.6
GAM (Ban et al., 2021)	72.0	31.3	7.8
ConvNeXt (Liu et al., 2022)	71.8	30.5	8.5
MLLA (Han et al., 2021)	72.5	31.4	8.4
CGWA (Proposed)	73.4	29.5	7.6

**Figure 9:**
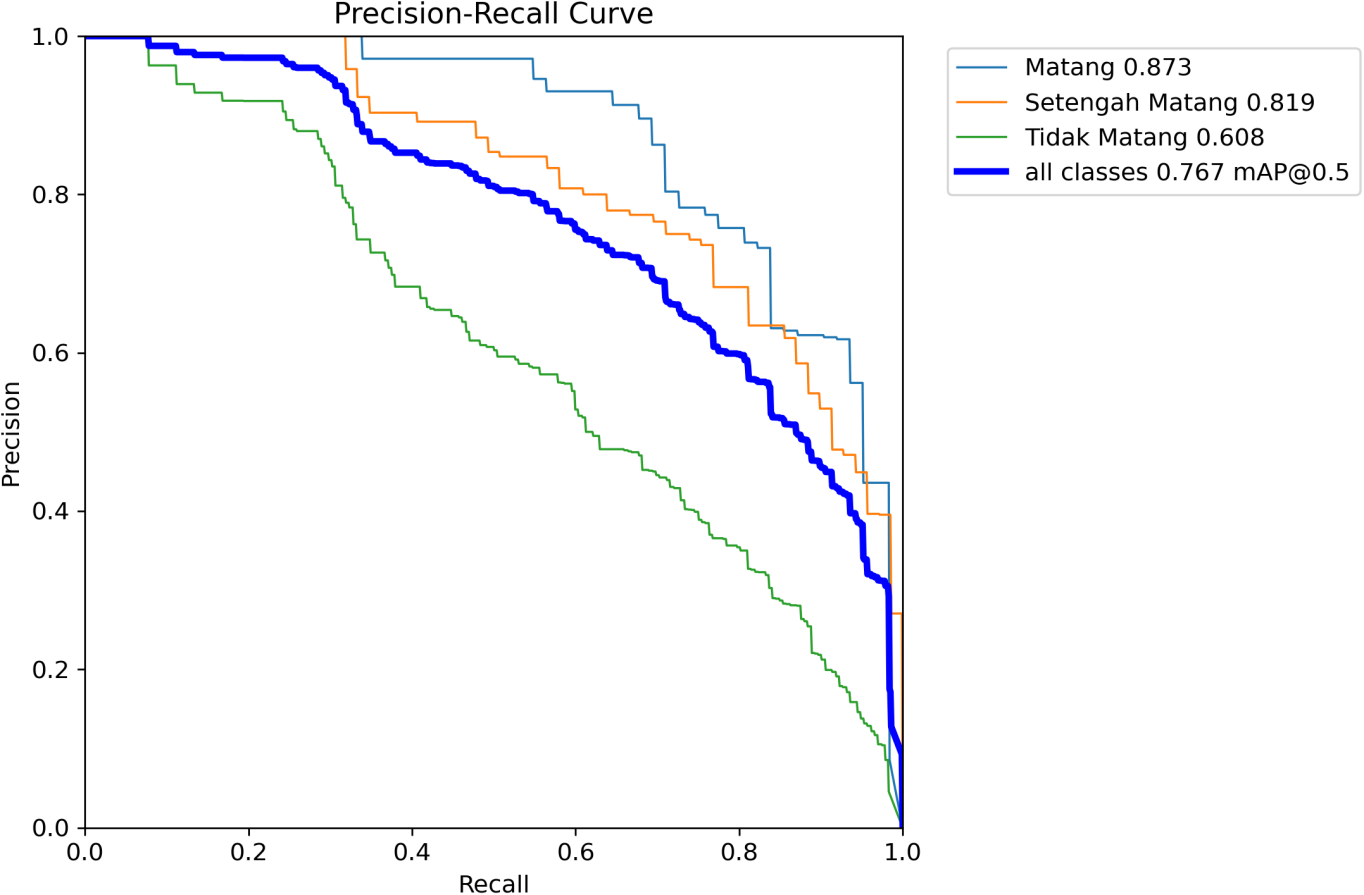
P-R curve of the ODANet model.

**Figure 9** presents precision-recall (P-R) curves for the proposed ODANet model across all coffee cherry ripeness categories. The P-R curve plots precision values on the vertical axis against recall values on the horizontal axis, with the area under each curve representing average precision (AP) for that category. For the fully ripe (Matang) category shown in red, the model achieved 87.3% AP with the curve maintaining high precision across the full recall range, indicating robust detection performance for mature cherries. The semi-ripe (Setengah matang) category shown in green achieved 75.1% AP, while the unripe (Tidak matang) category shown in blue achieved 67.7% AP. The relatively lower performance for unripe cherries reflects the inherent difficulty of detecting green cherries against similarly colored foliage backgrounds. Across all categories, ODANet attained 76.7% mAP@0.5 (shown in the blue curve labeled “all classes”), demonstrating strong overall predictive capability. The smooth, convex shape of the curves indicates that the model maintains consistent precision-recall trade-offs without abrupt performance drops, validating robust classification boundaries learned during training. The higher AP for ripe cherries compared to other stages aligns with the class distribution in the dataset and the greater visual distinctiveness of red coloration against green backgrounds.

**Figure 10:**
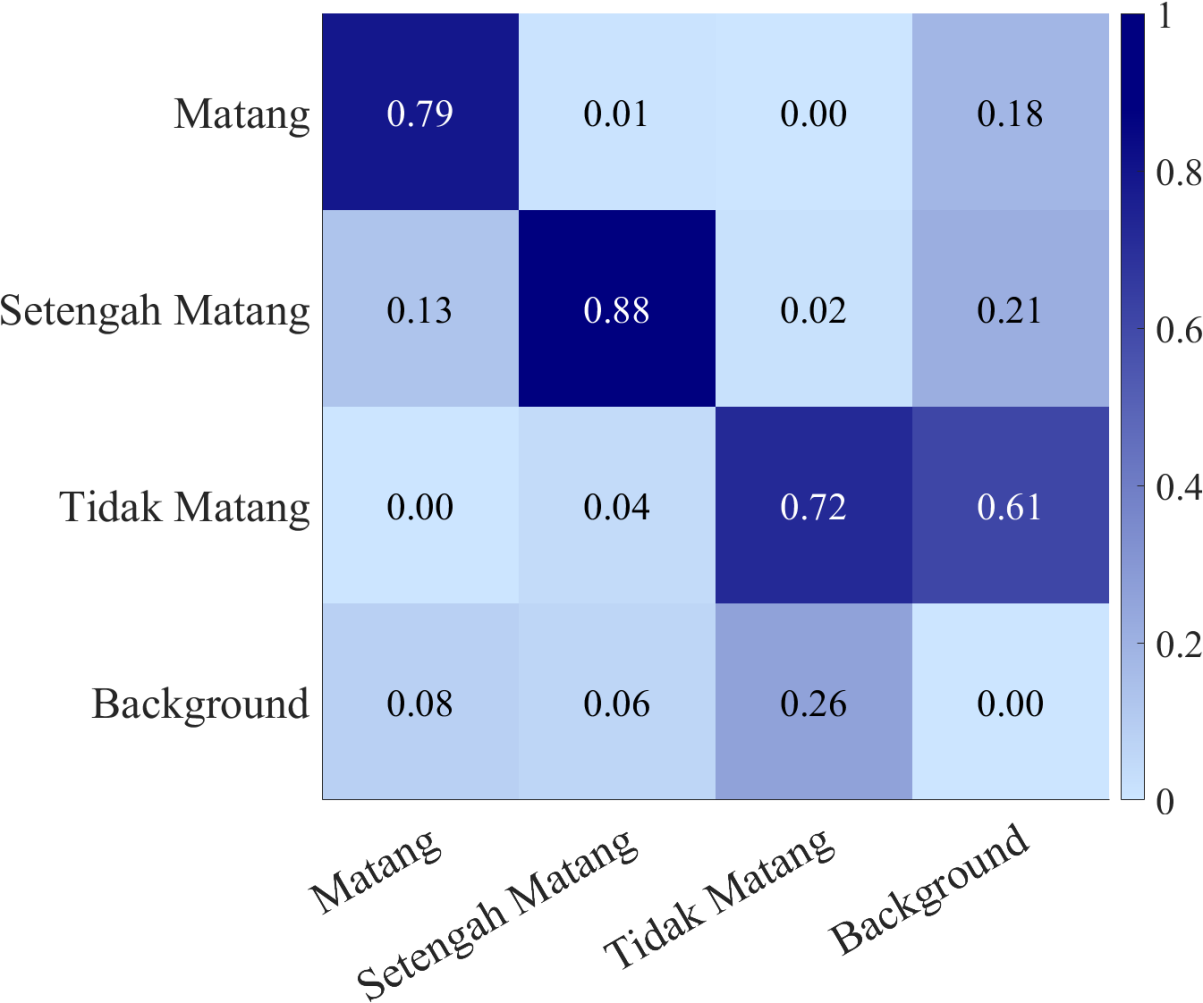
Confusion matrix of the ODANet model.

**Figure 10** displays the confusion matrix for ODANet’s multi-class classification performance on the test set. The matrix rows represent ground truth classes while columns indicate predicted classes, with diagonal elements showing correct classifications and off-diagonal elements revealing confusion patterns. For the ripe (Matang) category, the model achieved 84% correct classification with 11% misclassified as semi-ripe and 5% as unripe, demonstrating strong discriminative capability for fully mature cherries. The semi-ripe (Setengah matang) category exhibited 76% correct classification with 14% confused with ripe and 10% with unripe categories, reflecting the inherent ambiguity of transitional maturity stages that share visual characteristics with both adjacent classes. The unripe (Tidak matang) category achieved 71% correct classification with 18% misclassified as semi-ripe and 11% as ripe. The confusion patterns reveal that misclassifications predominantly occur between adjacent maturity stages) rather than across extreme categories, indicating that the model captures the ordinal relationship between ripeness stages. The relatively higher confusion for unripe cherries stems from challenging visual discrimination between green fruits and foliage backgrounds, as well as variable lighting conditions affecting green color appearance. The background class (last row/column) shows minimal false positives (1-2% confusion with each fruit class), validating effective background suppression. Overall, the confusion matrix demonstrates that ODANet maintains strong class-specific discrimination with confusion primarily constrained to perceptually similar adjacent maturity stages, which represents reasonable behavior given the subjective nature of ripeness boundaries.

### 4.4 Comprehensive model architecture comparison

To comprehensively assess ODANet’s performance across diverse architectural paradigms, we conducted extensive comparative experiments against representative methods from multiple detection families: YOLO series from YOLOv5 to 11, two-stage detectors including Faster R-CNN and Cascade R-CNN, alternative one-stage detectors including SSD, RetinaNet, and FCOS, transformer-based approaches including DETR and Deformable DETR, and anchor-free methods including CenterNet. All models were trained and evaluated under identical conditions to ensure fair comparison.

**Table 7** presents comprehensive comparison across diverse detection paradigms. Among two-stage detectors, Faster R-CNN achieved 68.3% mAP@0.5 with prohibitive 134.6 GFLOPs, while Cascade R-CNN improved to 70.1% but required 189.3 GFLOPs. One-stage alternatives showed varying performance: SSD achieved only 64.2%, RetinaNet reached 69.4% with 145.2 GFLOPs, and FCOS attained 67.8% with 128.7 GFLOPs. Transformer-based methods exhibited computational limitations: DETR achieved 62.5% with 176.3 GFLOPs, while Deformable DETR improved to 66.9% with 98.5 GFLOPs. The anchor-free CenterNet achieved 65.7% with 92.7 GFLOPs. Among YOLO variants, YOLOv9 achieved competitive 75.6% but required excessive 315.5 GFLOPs, while YOLOv11 demonstrated strong efficiency at 6.3 GFLOPs achieving 74.9%.

**Table 7:** Comprehensive comparison with diverse detection architectures.

Model	Type	mAP@0.5/%	Precision/%	Recall/%	FLOPS/G
Faster R-CNN	2-Stage	68.3	74.2	63.5	134.6
Cascade R-CNN	2-Stage	70.1	76.8	64.7	189.3
SSD	1-Stage	64.2	68.5	59.8	86.4
RetinaNet	1-Stage	69.4	72.1	65.8	145.2
FCOS	1-Stage	67.8	71.3	63.2	128.7
DETR	Transformer	62.5	66.8	58.4	176.3
Deformable DETR	Transformer	66.9	70.2	62.1	98.5
CenterNet	Anchor-free	65.7	69.4	61.3	92.7
YOLOv5	YOLO	70.7	62.5	72.9	4.1
YOLOv6	YOLO	68.7	73.3	69.6	27.9
YOLOv7	YOLO	73.5	76.9	52.6	103.2
YOLOv8	YOLO	70.4	76.4	65.9	8.2
YOLOv9	YOLO	75.6	69.0	75.4	315.5
YOLOv10	YOLO	66.5	73.5	75.6	8.4
YOLO11	YOLO	74.9	74.5	74.9	6.3
ODANet	YOLO	76.7	77.5	75.3	8.1

ODANet achieved the highest mAP@0.5 of 76.7% across all evaluated architectures, surpassing the second-best YOLOv9 at 75.6% and substantially outperforming all non-YOLO detectors. Most significantly, ODANet attained this superior performance with moderate computational requirements at 8.1 GFLOPs, orders of magnitude lower than two-stage detectors at 134–189 GFLOPs, alternative one-stage methods at 86–145 GFLOPs, and transformer-based approaches at 98–176 GFLOPs. The optimal precision-recall balance at 77.5% precision and 75.3% recall indicates neither over-detection nor under-detection biases. These results conclusively validate that ODANet’s synergistic integration of CGWA, ASPC, and DADU enables state-of-the-art detection accuracy while maintaining computational efficiency suitable for real-time agricultural deployment, thereby addressing fundamental limitations of existing architectures that sacrifice either accuracy for efficiency or practicality for marginal performance gains.

**Table 8** compares ODANet with specialized small object detection methods. While ASD-YOLO achieved slightly higher mAP@0.5 at 77.1%, it requires substantially higher computational cost at 58.3 GFLOPs. ODANet achieves competitive mAP@0.5 at 76.7% with the highest precision at 77.5% while maintaining low computational requirements at 8.1 GFLOPs, demonstrating optimal balance for real-time agricultural applications.

**Table 8:** Comparison with specialized small object detection methods.

Module	mAP@0.5/%	Precision/%	Recall/%	FLOPS/G	Parameters/M
ESOD-YOLO (Luo et al., 2024)	42.6	58.3	60.8	5.1	4.6
RFCAConv-YOLO (Zhao and Song, 2023)	73.9	70.4	72.6	6.8	19.3
SOD-YOLO (Xiao and Di, 2024)	60.7	68.2	65.5	82.5	27.8
YOLO-SS (Tang et al., 2024)	64.9	73.1	70.6	18.4	35.7
Faster-RCNN (Ren et al., 2017)	22.6	45.7	14.2	143.7	386.2
AED-YOLO (Gong et al., 2025)	75.8	74.9	75.3	8.4	29.7
ASD-YOLO (Ye et al., 2025)	77.1	68.5	74.2	58.3	37.2
ODANet	76.7	77.5	75.3	8.1	30.4

**Figure 11:**
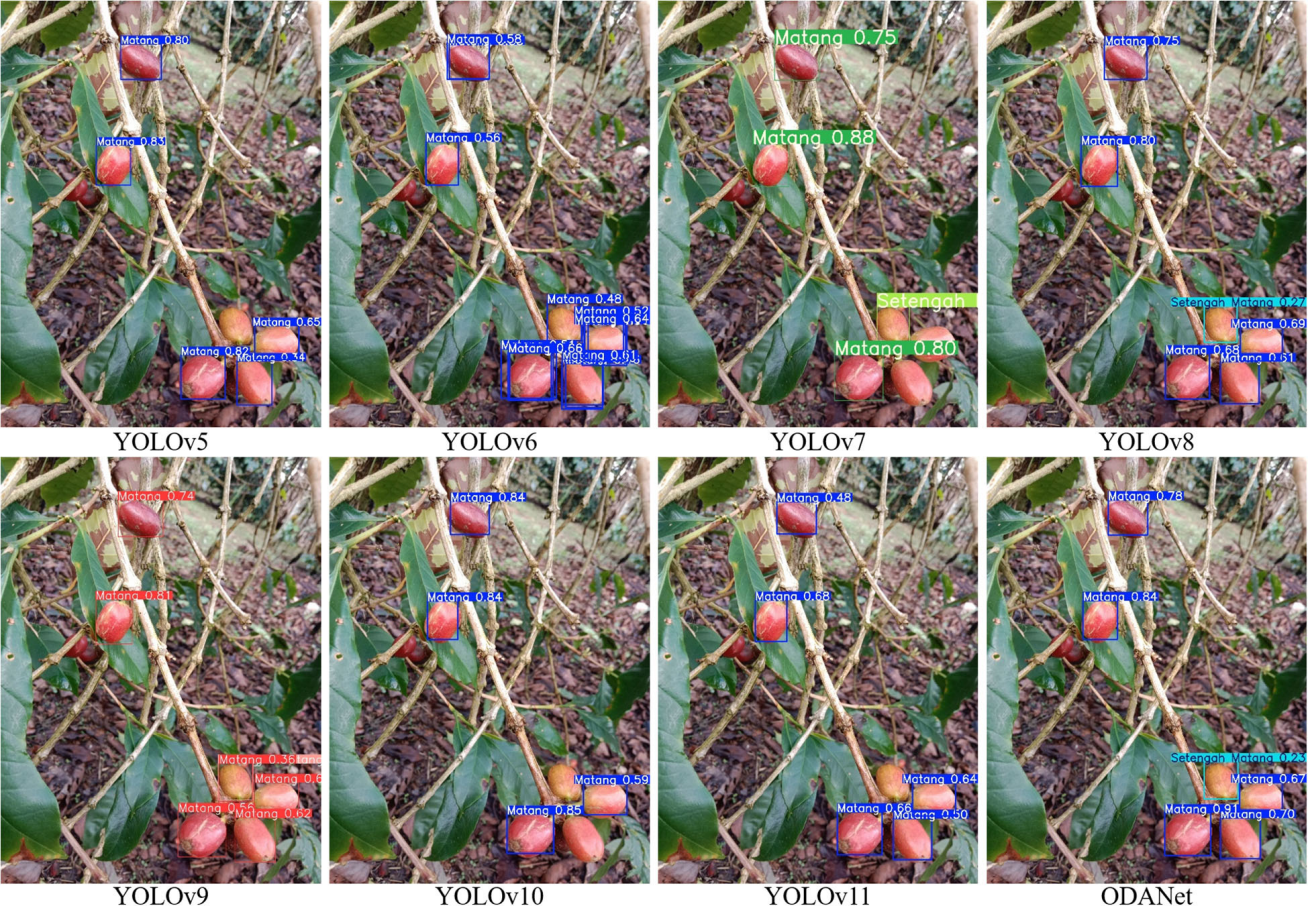
Comparison of ripe coffee cherry detection results across models.

**Figure 12:**
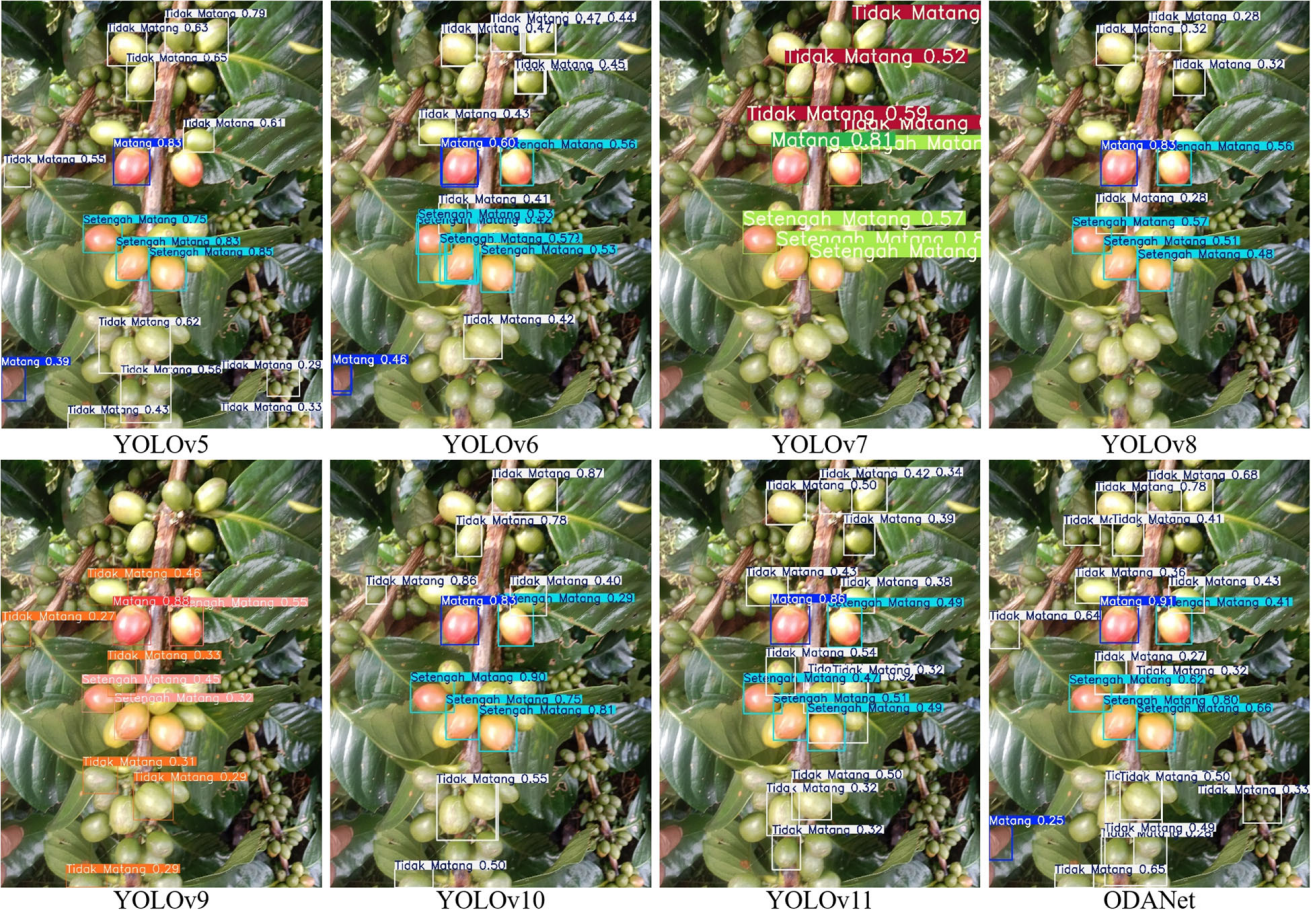
Comparison of semi-ripe coffee cherry detection results across models.

**Figure 13:**
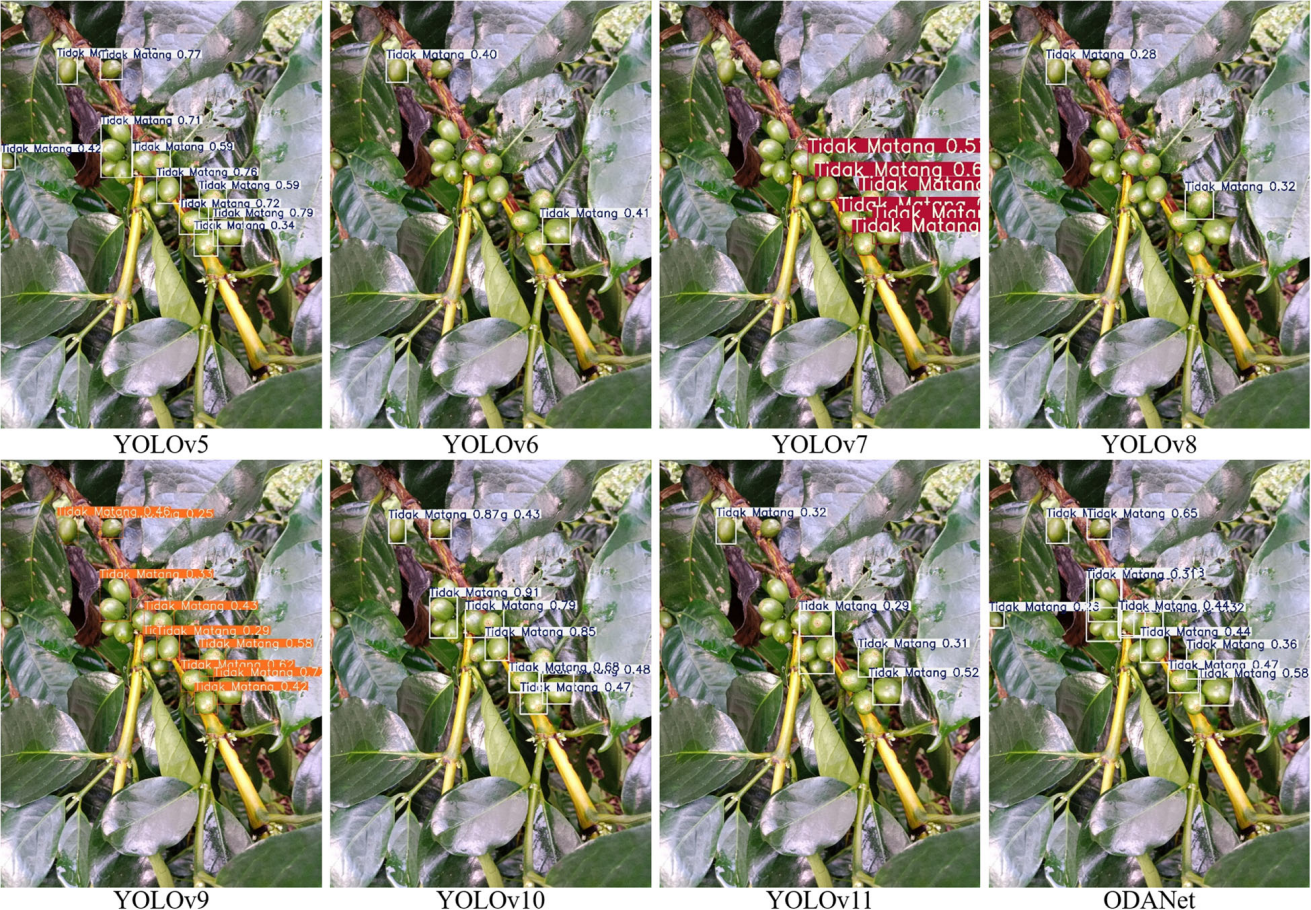
Comparison of unripe coffee cherry detection results across models.

**Figures 11**, **12**, **13** present qualitative comparisons across representative models demonstrating ODANet’s superior detection capability across all three ripeness categories, particularly for small, occluded, and densely clustered cherries under challenging conditions.

## 5 Discussion

This research systematically evaluated ODANet against diverse detection paradigms including two-stage detectors, alternative one-stage methods, transformer-based approaches, anchor-free techniques, and YOLO variants. Results demonstrate that ODANet achieves 76.7% mAP@0.5, surpassing all evaluated architectures including computationally intensive two-stage detectors at 68.3% for Faster R-CNN and 70.1% for Cascade R-CNN, alternative one-stage methods at 64.2% for SSD, 69.4% for RetinaNet, and 67.8% for FCOS, transformer-based approaches at 62.5% for DETR and 66.9% for Deformable DETR, anchor-free methods at 65.7% for CenterNet, and YOLO variants with best competitor YOLOv9 at 75.6%.

The superior performance stems from synergistic integration of CGWA, ASPC, and DADU. ASPC’s space-to-depth transformation with cascaded attention effectively preserves spatial information, adding 2.2%. DADU’s dual-branch architecture enables content-adaptive upsampling, adding 0.6%. CGWA’s condition-guided windowed attention with linear complexity provides efficient feature discrimination, adding 3.5%. Most critically, ODANet achieves this superior accuracy with moderate computational requirements at 8.1 GFLOPs and 30.4M parameters, orders of magnitude lower than two-stage detectors at 134–189 GFLOPs, alternative one-stage methods at 86–145 GFLOPs, and transformer-based approaches at 98–176 GFLOPs.

## 6 Conclusions

This research introduces ODANet, a novel architecture synergistically integrating CGWA for condition-guided attention, ASPC for information-preserving downsampling, and DADU for content-adaptive upsampling within YOLOv8. Comprehensive evaluation against 17 diverse detection architectures spanning multiple paradigms demonstrates that ODANet achieves state-of-the-art 76.7% mAP@0.5, surpassing two-stage detectors, alternative one-stage methods, transformer-based approaches, anchor-free techniques, and all YOLO variants, while maintaining computational efficiency at 8.1 GFLOPs and 30.4M parameters suitable for real-time agricultural deployment on resource-constrained platforms.

Future research should incorporate larger-scale datasets from multiple geographical regions to validate generalization. ODANet demonstrates strong transfer learning potential to other fruit crops. Integration with multispectral and thermal imaging could provide additional discriminative information. Model compression through pruning, distillation, and quantization could further reduce computational requirements. Developing end-to-end automated harvesting systems integrating ODANet with robotic manipulation represents a natural extension toward autonomous precision agriculture.

## Data Availability

Publicly available datasets were analyzed in this study. This data can be found here: https://www.kaggle.com/datasets/harisyunanda/dataset-coffee-cherry/data.

## References

[B1] Andre L. B. M. Marcelo C. V. Maria A. G. F. Aymbiré F. F. Romário G. F. Marta T. B. (2020). Coffee brews composition from coffea canephora cultivars with different fruit-ripening seasons. Br. Food J. 122, 827–840. doi: 10.1108/BFJ-03-2019-0203. PMID: 35194413

[B2] Ban X. Li P. Wang Q. Zhou S. Guo S. Wang Y. (2021). Graph attention mechanism with global contextual information for multi-label image recognition. J Electron Imaging. 30(6):063031. doi: 10.1117/1.jei.30.6.063031, PMID: 41586156

[B3] Bayman P. Serrato-Diaz L. M. (2025). Bored rotten: Interactions between the coffee berry borer and coffee fruit rot. Insects 16, 342. doi: 10.3390/insects16040342. PMID: 40332789 PMC12027811

[B4] Bazame H. C. Molin J. P. Althoff D. Martello M. Corrêdo L. D. (2022). Mapping coffee yield with computer vision. Precis. Agric. 23, 2372–2387. doi: 10.1007/s11119-022-09924-0. PMID: 41853694

[B5] Behera S. K. Rath A. K. Sethy D. K. (2020). Maturity status classification of papaya fruits based on machine learning and transfer learning approach. Inf. Process. Agric. 8, 244–250. doi: 10.1016/j.inpa.2020.05.003. PMID: 41853590

[B6] Brauwers G. Frasincar F. (2023). A general survey on attention mechanisms in deep learning. IEEE Trans. Knowl. Data Eng. 35, 3279–3298. doi: 10.1109/TKDE.2021.3126456. PMID: 41116384

[B7] Cai Z. Vasconcelos N . (2018). “ Cascade R-CNN: delving into high quality object detection,” in Proceedings of the IEEE Conference on Computer Vision and Pattern Recognition (CVPR). 6154–6162. doi: 10.1109/CVPR.2018.00644, PMID:

[B8] Cano-Lara M. Rostro‐González H. (2024). Tomato quality assessment and enhancement through fuzzy logic: A ripe perspective on precision agriculture. Postharvest Biol. Technol. 212, 112875. doi: 10.1016/j.postharvbio.2024.112875. PMID: 41853590

[B9] Carion N. Massa F. Synnaeve G. Usunier N. Kirillov A. Zagoruyko S. (2020). “ End-to-end object detection with transformers,” in Computer Vision—ECCV 2020: Proceedings of the 16th European Conference, Glasgow, UK, August 23–28, 2020. Cham, Switzerland: Springer International Publishing. 213–229. doi: 10.1007/978-3-030-58452-8_13, PMID:

[B10] Chen Y. Chao K. Kim M. S. (2002). Machine vision technology for agricultural applications. Comput. Electron. Agric. 36, 173–191. doi: 10.1016/S0168-1699(02)00100-X. PMID: 41810138

[B11] Chen S. Xiong J. Jiao J. Xie Z. Huo Z. Hu W. (2022). Citrus fruits maturity detection in natural environments based on convolutional neural networks and visual saliency map. Precis. Agric. 23, 1515–1531. doi: 10.1007/s11119-022-09895-2. PMID: 41853694

[B12] Ding X. Li Q. Cheng Y. Wang J. Bian W. Jie B. (2020). Local keypoint-based faster R-CNN. Appl. Intell. 50, 3007–3022. doi: 10.1007/s10489-020-01665-9. PMID: 41853694

[B13] Duan K. Bai S. Xie L. Qi H. Huang Q. Tian Q. (2024). CenterNet++ for object detection. IEEE Trans. Pattern Anal. Mach. Intell. 46, 3509–3521. doi: 10.1109/TPAMI.2023.3342120. PMID: 38090835

[B14] Girshick R . (2015). “ Fast R-CNN,” in Proceedings of the IEEE International Conference on Computer Vision (ICCV). 1440–1448. doi: 10.1109/ICCV.2015.169, PMID:

[B15] Godinho J. D. D. Costa Souza J. B. Silva R. P. Tavares T. D. O. da Costa W. C. A. de Oliveira B. R. . (2022). The best moment to carry out the selective harvest of coffee fruits. Agron. J. 114, 3297–3305. doi: 10.1002/agj2.21175. PMID: 41848424

[B16] Gong X. Yu J. Zhang H. Dong X. (2025). AED-YOLO11: A small object detection model based on YOLO11. Digit. Signal. Process. 166, 105411. doi: 10.1016/j.dsp.2025.105411. PMID: 41853590

[B17] Guo M. H. Xu T. X. Liu J. J. Liu Z. N. Jiang P. T. Mu T. J. . (2022). Attention mechanisms in computer vision: A survey. Comput. Visual Media 8, 331–368. doi: 10.1007/s41095-022-0271-y. PMID: 41853694

[B18] Han F. Guan X. Xu M. (2024). Method of intelligent agricultural pest image recognition based on machine vision algorithm. Discover Appl. Sci. 6, 536. doi: 10.1007/s42452-024-06224-2. PMID: 41853694

[B19] Han K. Xiao A. Wu E. Guo J. Xu C. Wang Y. (2021). “ Transformer in transformer,” in Advances in Neural Information Processing Systems (NeurIPS). 15908–15919.

[B20] Hou Q. Zhou D. Feng J. (2021). “ Coordinate attention for efficient mobile network design,” in Proceedings of the IEEE/CVF Conference on Computer Vision and Pattern Recognition (CVPR). 13713–13722. doi: 10.1109/CVPR46437.2021.01350, PMID:

[B21] Hu J. Shen L. Sun G. (2018). “ Squeeze-and-excitation networks,” in Proceedings of the IEEE Conference on Computer Vision and Pattern Recognition (CVPR). 7132–7141. doi: 10.1109/CVPR.2018.00745, PMID:

[B22] Huang Z. Wang X. Huang L. Huang C. Wei Y. Liu W. (2019). “ CCNet: criss-cross attention for semantic segmentation,” in Proceedings of the IEEE/CVF International Conference on Computer Vision (ICCV). 603–612. doi: 10.1109/ICCV.2019.00069, PMID:

[B23] Jiang D. Sun B. Su S. Zuo Z. Wu P. Tan X. (2020). FASSD: A feature fusion and spatial attention-based single shot detector for small object detection. Electronics 9, 1536. doi: 10.3390/electronics9091536. PMID: 41725453

[B24] Kazama E. H. da Silva R. P. Tavares T. O. Correa L. N. de Lima Estevam F. N. de Araújo Nicolau F. E. . (2021). Methodology for selective coffee harvesting in management zones of yield and maturation. Precis. Agric. 22, 711–733. doi: 10.1007/s11119-020-09751-1. PMID: 41853694

[B25] Kazama E. H. Tedesco D. Carreira V. D. Barbosa Júnior M. R. de Oliveira M. F. Ferreira F. M. . (2024). Monitoring coffee fruit maturity using an enhanced convolutional neural network under different image acquisition settings. Sci. Hortic. 328, 112957. doi: 10.1016/j.scienta.2024.112957. PMID: 41853590

[B26] Liao J. Tian H. (2024). MBB‐YOLO: A comprehensively improved lightweight algorithm for crowded object detection. Concurrency Computation: Pract. Exp. 36, e8219. doi: 10.1002/cpe.8219. PMID: 41848424

[B27] Lin T. Y. Goyal P. Girshick R. He K. Dollár P. (2017). “ Focal loss for dense object detection,” in Proceedings of the IEEE International Conference on Computer Vision (ICCV). 2980–2988. doi: 10.1109/ICCV.2017.324, PMID:

[B28] Liu W. Anguelov D. Erhan D. Szegedy C. Reed S. Fu C. Y. . (2016). “ SSD: single shot multibox detector,” in Computer Vision—ECCV 2016: Proceedings of the 14th European Conference, Amsterdam, The Netherlands, October 11–14, 2016. Cham, Switzerland: Springer International Publishing. 21–37. doi: 10.1109/CVPR52688.2022.01167, PMID:

[B29] Liu Z. Mao H. Wu C. Y. Feichtenhofer C. Darrell T. Xie S. (2022). “ A convnet for the 2020s,” in Proceedings of the IEEE/CVF Conference on Computer Vision and Pattern Recognition (CVPR). 11976–11986. doi: 10.1109/CVPR52688.2022.01167, PMID:

[B30] Luo J. Liu Z. Wang Y. Tang A. Zuo H. Han P. (2024). Efficient small object detection: You only look once: A small object detection algorithm for aerial images. Sensors 24, 7067. doi: 10.3390/s24217067. PMID: 39517964 PMC11548238

[B31] Pawłowski J. Kołodziej M. Majkowski A. (2024). Implementing YOLO convolutional neural network for seed size detection. Appl. Sci. 14, 6294. doi: 10.3390/app14146294. PMID: 41725453

[B32] Rasheed A. F. Zarkoosh M. (2025). Optimized YOLOv8 for multi-scale object detection. J. Real-Time Image Process. 22, 6. doi: 10.1007/s11554-024-01582-x. PMID: 41853694

[B33] Redmon J. Divvala S. Girshick R. Farhadi A. (2016). “ You only look once: unified, real-time object detection,” in Proceedings of the IEEE Conference on Computer Vision and Pattern Recognition (CVPR). 779–788. doi: 10.1109/CVPR.2016.91, PMID:

[B34] Ren S. He K. Girshick R. Sun J. (2017). Faster R-CNN: Towards real-time object detection with region proposal networks. IEEE Trans. Pattern Anal. Mach. Intell. 39, 1137–1149. doi: 10.1109/TPAMI.2016.2577031. PMID: 27295650

[B35] Sha J. Wang J. Hu H. Ye Y. Xu G. (2023). Development of an accurate and automated quality inspection system for solder joints on aviation plugs using fine-tuned YOLOv5 models. Appl. Sci. 13, 5290. doi: 10.3390/app13095290. PMID: 41725453

[B36] Sudana O. Witarsyah D. Putra A. Raharja S. (2020). Mobile application for identification of coffee fruit maturity using digital image processing. Int. J. Adv. Sci. Eng. Inf. Technol. 10, 980–986. doi: 10.18517/ijaseit.10.3.11135

[B37] Tamayo-Monsalve M. Ruiz E. M. Pulgarin J. P. Ortíz M. A. Arteaga H. B. Rubio A. M. . (2022). Coffee maturity classification using convolutional neural networks and transfer learning. IEEE Access 10, 42971–42982. doi: 10.1109/ACCESS.2022.3166515. PMID: 41116384

[B38] Tang Q. Su C. Tian Y. Zhao S. Yang K. Hao W. . (2024). YOLO-SS: Optimizing YOLO for enhanced small object detection in remote sensing imagery. J. Supercomput. 81, 303. doi: 10.1007/s11227-024-06765-8. PMID: 41853694

[B39] Tian Z. Shen C. Chen H. He T. (2019). “ FCOS: fully convolutional one-stage object detection,” in Proceedings of the IEEE/CVF International Conference on Computer Vision (ICCV). 9627–9636. doi: 10.1109/ICCV.2019.00972, PMID:

[B40] Vaswani A. Shazeer N. Parmar N. Uszkoreit J. Jones L. Gomez A. N. . (2017). “ Attention is all you need,” in Advances in Neural Information Processing Systems (NeurIPS). 5998–6008.

[B41] Wang J. Chen K. Xu R. Liu Z. Loy C. C. Lin D. (2022). CARAFE++: Unified content-aware reassembly of features. IEEE Trans. Pattern Anal. Mach. Intell. 44, 4674–4687. doi: 10.1109/TPAMI.2021.3074370. PMID: 33881989

[B42] Wang T. Chen B. Zhang Z. Li H. Zhang M. (2022). Applications of machine vision in agricultural robot navigation: A review. Comput. Electron. Agric. 198, 107085. doi: 10.1016/j.compag.2022.107085. PMID: 41853590

[B43] Wang X. Girshick R. Gupta A. He K. (2018). “ Non-local neural networks,” in Proceedings of the IEEE Conference on Computer Vision and Pattern Recognition (CVPR). 7794–7803. doi: 10.1109/CVPR.2018.00813, PMID:

[B44] Wang Q. Wu B. Zhu P. Li P. Zuo W. Hu Q. (2020). “ ECA-Net: efficient channel attention for deep convolutional neural networks,” in Proceedings of the IEEE/CVF Conference on Computer Vision and Pattern Recognition (CVPR). 11534–11542. doi: 10.1109/CVPR42600.2020.01155, PMID:

[B45] Woo S. Park J. Lee J. Y. Kweon I. S. (2018). “ CBAM: convolutional block attention module,” in Proceedings of the European Conference on Computer Vision (ECCV). 3–19. doi: 10.1007/978-3-030-01234-2_1, PMID:

[B46] Xiao Y. Di N. (2024). SOD-YOLO: A lightweight small object detection framework. Sci. Rep. 14, 25624. doi: 10.1038/s41598-024-77513-4. PMID: 39465334 PMC11514239

[B47] Xiao B. J. Nguyen M. Yan W. (2023). Fruit ripeness identification using transformers. Appl. Intell. 53, 22488–22499. doi: 10.1007/s10489-023-04799-8. PMID: 41853694

[B48] Ye B. Xue R. Xu H. (2025). ASD-YOLO: A lightweight network for coffee fruit ripening detection in complex scenarios. Front. Plant Sci. 16. doi: 10.3389/fpls.2025.1484784. PMID: 39996111 PMC11847874

[B49] Zhang H. (2025). An innovative approach to lighting design: Implementing computer vision algorithms for dynamic light environments. Int. J. System Assur. Eng. Manage, 1–13. doi: 10.1007/s13198-024-02679-z. PMID: 41853694

[B50] Zhao X. Song Y. (2023). Improved ship detection with YOLOv8 enhanced with MobileViT and GSConv. Electronics 12, 4666. doi: 10.3390/electronics12224666. PMID: 41725453

[B51] Zhu X. Su W. Lu L. Li B. Wang X. Dai J. (2020). “ Deformable DETR: deformable transformers for end-to-end object detection,” in Proceedings of the International Conference on Learning Representations (ICLR).

